# High Throughput Sequencing and Network Analysis Disentangle the Microbial Communities of Ticks and Hosts Within and Between Ecosystems

**DOI:** 10.3389/fcimb.2018.00236

**Published:** 2018-07-09

**Authors:** Agustín Estrada-Peña, Alejandro Cabezas-Cruz, Thomas Pollet, Muriel Vayssier-Taussat, Jean-François Cosson

**Affiliations:** ^1^Faculty of Veterinary Medicine, University of Zaragoza, Zaragoza, Spain; ^2^UMR BIPAR, INRA, ANSES, Ecole Nationale Vétérinaire d'Alfort, Université Paris-Est, Maisons-Alfort, France

**Keywords:** microbiome, ticks, voles, network analysis, *Ixodes ricinus*, next generation sequencing (NGS)

## Abstract

We aimed to develop a framework, based on graph theory, to capture the ecological meaning behind pure pair comparisons of microbiome-derived data. As a proof of concept, we applied the framework to analyze the co-occurrence of bacteria in either *Ixodes ricinus* ticks or the spleen of one of their main hosts, the vole *Myodes glareolus*. As a secondary lymphoid organ, the spleen acts as a filter of blood and represents well the exposure to microorganisms circulating in the blood; including those acquired and transmitted by ticks during feeding. The microbiome of 301 and 269 individual tick and vole samples, respectively, were analyzed using next generation sequencing (NGS) of 16S rRNA. To assess the effect of habitat on ecological communities of bacteria associated to ticks and voles, two different biotopes were included in the study, forest, and ecotone. An innovative approach of NGS data analysis combining network analysis and phylogenies of co-occuring of bacteria was used to study associations between bacteria in individual samples. Of the 126 bacterial genera found in ticks and voles, 62% were shared by both species. Communities of co-occurring bacteria were always more phylogenetically diverse in ticks than in voles. Interestingly, ~80% of bacterial phylogenetic diversity was found in ~20% of ticks. This pattern was not observed in vole-associated bacteria. Results revealed that the microbiome of *I. ricinus* is only slightly related to that of *M. glareolus* and that the biotope plays the most important role in shaping the bacterial communities of either ticks or voles. The analysis of the phylogenetic signal of the network indexes across the 16S rRNA-derived tree of bacteria suggests that the microbiome of both ticks and voles has high phylogenetic diversity and that closest bacterial genera do not co-occur. This study shows that network analysis is a promising tool to unravel complex microbial communities associated to arthropod vectors and vertebrate hosts.

## Introduction

The term microbiome refers to the assemblage of bacteria that is found, either as symbiotic, commensal or pathogen in a living organism (Finney et al., 2015). Initial studies in ticks were aimed for detecting pathogenic microorganisms that could be transmitted to vertebrates during blood feeding. After a blood meal, the pathogen enters and colonizes the tick gut and subsequently migrates to the salivary glands from where it is transmitted to a new host. Once the tick is infected, the microorganism can be maintained through subsequent developmental stages of the tick (i.e., transstadial transmission) or pass to the next generation of ticks via transovarial transmission (Telford and Goethert, 2008). Bacterial assemblages within arthropod vectors attracted the attention of the researchers because they were found to influence pathogen ecology and transmission (Narasimhan et al., 2014; Finney et al., 2015; Abraham et al., 2017). For example, the presence of certain bacteria blocks the transmission of arboviruses by mosquitoes (Moreira et al., 2009). In the tick *Ixodes scapularis*, the microbiota composition influences *Borrelia burgdorferi* colonization of tick gut (Narasimhan et al., 2014). An inverse example showed that tick colonization by *Anaplasma phagocytophilum* perturbs the gut microbiota (Abraham et al., 2017).

In the last years, studies aiming to identify tick-borne pathogens assessed only minimal numbers of species at a time, partly due to technological limitations. Recent advances in DNA sequencing technologies have increased our ability to characterize large numbers of tick-associated microorganisms at a time (Nakao et al., 2013; Zhang et al., 2014). While pioneering studies have improved our understanding of the diversity of tick-associated microorganisms, a profusion of bacterial species has been found in ticks (Carpi et al., 2011; Nakao et al., 2013; Zhang et al., 2014; Van Treuren et al., 2015; Sui et al., 2017; Swei and Kwan, 2017). The generation of large datasets in next generation sequencing projects faces the challenge of data analysis to answer relevant questions such as how these microbial communities are organized. It is thus paramount to develop methods to capture the microbial community-level patterns (Kautz et al., 2013). Network analysis (or graph theory) offers the methodological framework to produce simple indexes that ideally define the associations, but not the trophic interactions, of each microorganism in the structure of the microbiome.

This study is aimed to build a network-based framework for analyzing co-occurrence patterns of microorganisms in *Ixodes ricinus* ticks and one of its main hosts, the vole *Myodes glareolus*. The focus is neither on the description of the ticks or vole microbiomes or the differences in bacterial composition between tick instars. The proposed framework is a proof of concept that uses a large dataset of bacterial genera of both ticks and voles recorded at two different ecosystems. We intended to provide a foundation for analyzing hierarchical levels of complexity in the tick microbiome, phylogenetic relationships among bacteria of ticks and voles, yielding an example with immediate application to similar cases.

## Materials and methods

### Ethics statement

Animals were treated in accordance with European Union guidelines and legislation (Directive 86/609/EEC). Sampling of rodents, storage, and use of their tissues were conducted under the approval (no. B 34-169-003), from the Departmental Direction of Population Protection (DDPP, Hérault, France). The species investigated in this study has no protected status.

### Surveys

Tick and rodent samplings were conducted in the French Ardennes as previously described (Guivier et al., 2011). Briefly, we sampled six forested sites along a transect line of approximately 80 km, and three ecotones (i.e., edge networks within open grasslands) for both ticks and rodents. Each site corresponded to an area of about 2 km2 and the minimum distance between sites was 3.2 km (Guivier et al., 2011). A total of 177 voles were collected in the forest sites (92 ♂, 84 ♀) and 92 voles were collected in the ecotone sites (42 ♂, 50 ♀). Rodents were dissected following Herbreteau et al. (2011) to avoid environmental and cross-samples bacterial DNA contamination. Spleens of voles were selected as target organs, placed in RNAlater (Sigma, St. Louis, MO, United States) and stored at −20°C. The spleen, a secondary lymphoid organ, acts as a filter of blood and therefore is a good “proxy” of exposure to microorganisms circulating in the blood; including those acquired and transmitted by ticks during feeding. Questing *Ixodes ricinus* adult ticks were collected by flagging, surface sterilized, individually crushed and stored at −20°C, as previously described (Moutailler et al., 2016). A total of 228 ticks were collected in forest sites (28 ♂, 199 ♀) and 73 in the ecotone (4 ♂, 69 ♀).

### DNA preparation

In order to avoid cross-contamination of samples with environmental bacterial DNA, pre-PCR laboratory manipulations were conducted with filter tips under a sterile hood in a DNA-free room. DNA was extracted with the DNeasy 96 Tissue Kit (Qiagen, Hilden, Germany) with final elution in 200 μl of elution buffer. One extraction blank, corresponding to an extraction without sample tissue, was systematically added to each of the DNA extraction microplates. DNA was quantified with a NanoDrop 8000 spectrophotometer (Thermo Scientific, Waltham, MA, United States), to confirm the presence of a minimum of 10 ng/μl of DNA in each sample.

### Bacterial screening

Bacterial screening followed the protocol of Galan et al. (2016). Briefly, bacteria were detected using a 251-bp fragment of the 16S rRNA gene V4 region, known to have a good accuracy for genus assignation. All samples were tagged using a unique combination of indexes during PCR amplification, then pooled and sequenced in an Illumina Miseq run. The tagging allows assigning each sequence read to a unique sample. Positive and negative controls were added at different steps of the laboratory manipulations to detect potential contaminations across samples and by laboratory reagents. Each sample was systematically analyzed into two separate replicates, from DNA amplification to the end of the process.

### High-throughput sequencing data analyses and taxonomic classification of OTUs

We use rigorous experimental procedures for the direct estimation of biases from the data produced by 16S rRNA amplicon sequencing (Galan et al., 2016). MiSeq datasets were processed with mothur v1.34 (Schloss et al., 2009) and with the MiSeq standard operating procedure (Kozich et al., 2013). This allowed us to: (1) construct contigs of paired-end read 1 and read 2 using the make.contig command; (2) remove the reads with poor quality of assembly (> 275 bp); (3) align unique sequences on the SILVA SSU Reference alignment v119 (Quast et al., 2013); (4) remove the misaligned, non-specific (eukaryotic) and chimeric reads (uchime program); (5) regroup the reads into Operational Taxonomic Units (OTUs) with a 3% divergence threshold; and (6) classify the OTUs using the Bayesian classifier included in mothur (bootstrap cutoff = 80%) and the Silva taxonomic file. As mentioned above, we made PCR replicates for each rodent sample and several controls were included in each sequencing run. Therefore, we had more PCR products than rodent samples. We obtained a table with the number of reads for each OTU (rows) and each PCR product (columns).

The stepwise trimming process described in Galan et al. (2016) was then applied to clean the raw dataset from bacterial contaminations and false assignation of reads to samples. Sample replicates were checked for consistency and samples were considered to be positive for a given OTU only if both PCR replicates represent positive results. Sample replicates positive for given OTU were combined by adding read numbers for each OTU. The completion of bacteria catalogs for each sample was evaluated using cumulative curves (taxa number *vs*. reads number) using the Past Software v3.15 (Hammer et al., 2001). Furthermore, the taxonomic identification of the OTUs was refined through phylogenetic and blast analyses. As previously described (Galan et al., 2016), these steps allowed us to reach a highly accurate taxonomic classification to genus level. Table S1 provides a complete overview of OUT's, genera, and number of reads for each individual vole or tick, collected in in either forest or ecotone.

### Phylogenetic analysis

The 16S rDNA reads length resulting from our High-Throughput Sequencing (HTS) were too small (251-bp) to build a reliable phylogenetic tree. To overcome this issue, 16S rDNA nucleotide sequences of 126 bacteria species (one bacteria species representing each bacterial genus, available as Tables S2, S3, Figure S1) were obtained from GenBank. This approach is based on the reasonable assumption that bacteria species within a genus are more related between them than to any other bacteria of other genera. Sequences were aligned using MAFFT (Katoh and Standley, 2013). The evolutionary history was inferred using the Neighbor-Joining method (Saitou and Nei, 1987), implemented in MEGA 6. Reliability of internal branches was assessed using the bootstrapping method (1,000 replicates). The evolutionary distances were computed using the Maximum Composite Likelihood method (Tamura et al., 2004). The rate variation among sites was modeled with a gamma distribution (shape parameter = 0.64). All positions containing gaps and missing data were eliminated. This phylogenetic tree was used to test the phylogenetic niche conservatism of the bacterial genera. Phylogenetic conservatism occurs when closely related taxa appear together more frequently than by pure chance. We tested if there were significant associations between prevalence of the bacteria in ticks/voles or forest/ecotone and phylogenetic distances (PD, calculated after Faith, 1992) using Pagel's λ (Pagel, 1999). A λ near 0 indicates very little phylogenetic signal in the trait data (prevalence) given the phylogenetic tree and a λ near 1 indicates relatively more phylogenetic signal given the phylogenetic tree. The ecological meaning of this test is to check if phylogenetically close genera of bacteria tend to appear together more frequently in the same carrier (tick or vole) or biotope (forest or ecotone).

### Building network relationships among bacteria

We explicitly propose a method based on the relationships of co-occurring bacteria using the basic tenets of the network theory, as a way to capture the communities in the studied microbiome. Networks are constructs reflecting the relationships between interacting partners: two bacteria are related by a link if they were simultaneously detected in a single carrier (tick or vole). We used the genera of bacteria because (i) using OTUs introduced considerable noise in the networks, impacting the analysis of the associations, and (ii) using taxa higher than genera obscured the associations. Figure S2 shows the flow of computations, of applicability to any situation where the co-occurrence among taxa is used to build a network. All the calculations were done on the software platform Gephi (gephi.org, accessed March, 2017) an open source software that transforms co-occurrence data and in a graph.

Four networks were separately built for each combination of carrier and biotope, using the number of times every pair of bacteria was detected for a combination. A set of rules was established to filter poorly represented genera: (i) we discarded the OTUs that were not assigned at the genus level; (ii) we removed the bacteria which appeared only once in the complete set of samples (singleton); and (iii) we established a cut-off value of reads to remove a microorganism. Occurrences with reads below the 10% of the frequency distribution of reads of a microorganism in the set of samples were removed, establishing an individual cut-off level for each microorganism.

The network-derived index of weighted degree (WD) of a microorganism results from its interactions with the other taxa and is the basis to calculate several indicators that rank the microorganisms in the network (Gómez et al., 2013; Estrada-Peña et al., 2015). We calculated the gamma exponent of the power-law distribution of WD, which explains if a network is scale-free (Barabási and Albert, 1999). The scale-free feature is a characteristic of natural networks, appearing when the genera of bacteria were used. When family of bacteria were used, the network lost the power-law distribution followed by scale-free networks. Therefore, only genera of bacteria were used in further analysis. We calculated the basic indexes ranking the importance of each bacterial genus with the network. Betweenness centrality (BNC) gives a higher score to a node (bacterial genus) that sits on many shortest paths between other node pairs (Newman, 2005). If the genera A and B appear frequently together, and the genera B and C are also tightly associated, then the genus B has a high BNC. The Clustering Coefficient (CC) of a node indicates more densely interconnections of its neighbors. For the matter of microbial (bacteria in this study) networks, a community is a set of co-occurring genera that cluster together more frequently than with the rest. We detected the communities of co-occurring bacteria within the microbiomes of ticks and voles using the algorithm of Newman (2004).

We further tested the phylogenetic signal of the network indexes on the genetic tree of bacteria using Pagel's λ as before. The aim was to check if some branches of the tree have a particular combination of network-derived indexes. We also computed the similarities between the microbiomes detected in carrier(s) and biotope(s), using the Sørensen index based on presence/absence data (Ludwig and Reynolds, 1988). This index varies from 0 (totally dissimilar) to 1 (complete similarity).

### Building network relationships among ticks and voles

We aimed to capture the relatedness among individual ticks and voles regarding the carried microorganisms. Carriers were individually related according to the similarity of co-occurring bacteria: each link between either two ticks, voles, or tick-vole was loaded with the number of shared bacterial genera. We calculated the communities of ticks and voles from the resulting networks according to Newman (2004), in both forest and ecotone. For each community, we computed the phylogenetic diversity of carried bacteria, as well as the percent of carriers and the number of bacterial genera circulated by each community.

### Host specificity of the recorded bacteria

We aimed to answer whether a microorganism was more commonly associated to either ticks or voles, or whether it was circulating among carriers without significant differences of detection in either ticks or voles. This discrimination has deep roots in ecological classifications, classically addressed using methods based on multivariate statistics (i.e., Lozupone et al., 2011; La Rosa et al., 2012). Our approach was based in comparing the WD of each microorganism and the ratio of the number of reads in either voles or ticks. The average number of reads of every microorganism was converted to log (0.1 + value) and the calculations were done separately for biotopes (forest and ecotone). It is assumed that a high ratio of the number of reads (voles/ticks or ticks/voles) plotted against the WD values will pinpoint the bacterial genera that are more frequently associated to either voles or ticks. Intermediate values would indicate bacteria that could circulate among voles and ticks without a clear component of specificity toward a given reservoir.

## Results

### Microbiome of ticks and voles

The samples from the 269 voles and the 301 ticks gave 4,749,786 reads meeting our quality control standards. Mean number of reads per sample was 8,193. Reads were assigned to 525 OTUs. Of these, 6 (1.14%) OTUs were singletons, and were not included in further analyses.

The resulting 519 OTUs represent 126 genera of bacteria, distributed in 79 Families and 47 Orders (Table S4). Mean number of bacterial genera identified in individual ticks and individual rodents were very similar (13.42 vs. 13.44, min. 2–1, max. 33–37, respectively). There was no positive correlation between the number of genera and the number of reads in individual samples (*R*^2^ = 0.0002), indicating no evidence of bias in the number of genera detected in a sample according to read numbers. Furthermore, rarefaction curves level off well before 5,000 reads (Figure S3) indicating that there was sufficient number of reads to draw a reliable list of bacterial genera within each ticks and voles.

**Figure 1:**
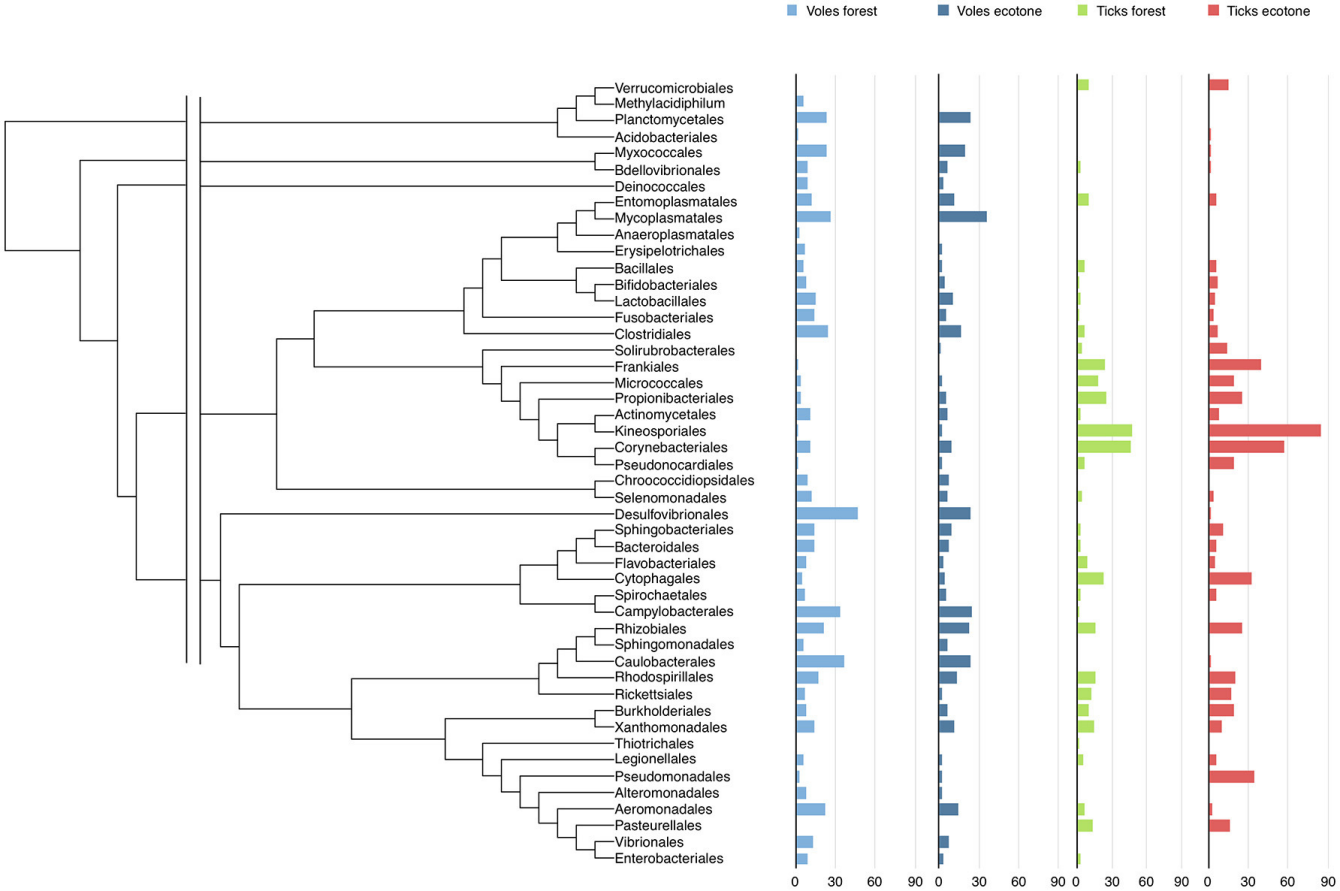
The phylogeny and the prevalence of the Orders of bacteria detected in voles-forest, voles-ecotone, ticks-forest, and ticks-ecotone. The complete data on prevalence according to the Genera of bacteria is included in Table S3. The fasta file including the *16S* rDNA sequences used to build the tree of genera, the nexus file for phylogenetic distances, and the phylogenetic tree of bacterial genera are included as Tables S2, S3 and Figure S2.

Tick and vole microbiomes shared 62% of bacterial genera. A total of 36 genera were detected only in voles. Most noticeable is the absence of *Helicobacter, Leptospira, Listeria, Mycoplasma, Orientia, Treponema*, and *Vibrio* in ticks. A total of 12 genera were recorded only in ticks. Most noticeable is the absence of *Luteolibacter, Knoellia, Wautersiella*, and *Frondihabitans* in voles. Prevalence of bacteria varied according to carriers and biotopes, both at the genus and at the other taxonomic levels (Figure 1, Table S4).

Figure S4 includes the frequency distribution of the number of bacterial genera recorded for ticks and voles, in either forest or ecotone. Figure S5 includes the frequency distribution of the number of reads recorded for ticks and voles, in either forest or ecotone. Bacterial assemblages of each carrier are remarkably dissimilar between voles and ticks. Sørensen index was 0.776 for voles in forest-ecotone, and 0.763 for ticks in forest-ecotone. However, Sørensen index was in the range 0.235–0.282 in both biotopes. Thus, the biotope has an impact in the outline of bacterial assemblages of either ticks or voles. The phylogenetic signal of bacterial prevalence is low (ticks-forest: 0.126; ticks-ecotone: 0.126; voles-forest: 0.053; voles-ecotone: 0.029, in the range 0–1). The interpretation is that genetically similar bacteria do not tend to occur together in any combination of carriers or biotopes.

### Properties of bacterial networks

Four networks were built on the co-occurrence patterns of bacterial genera associated to either ticks or voles in the forest or the ecotone. The network of ticks-forest has 85 nodes and 2,282 links, voles-forest has 109 nodes and 3,847 links, ticks-ecotone has 80 nodes and 1,950 links, and voles-ecotone has 100 nodes and 2,148 links (Figure S6, Table S5). The weights of every network follow a power-law distribution, meaning for a topology in “small world.” Most nodes are connected to neighbor nodes in every network, preventing the occurrence of large hubs. However, the power-law distribution is missing if higher taxonomic units (i.e., bacterial Families or Orders) are used to build the network.

**Figure 2:**
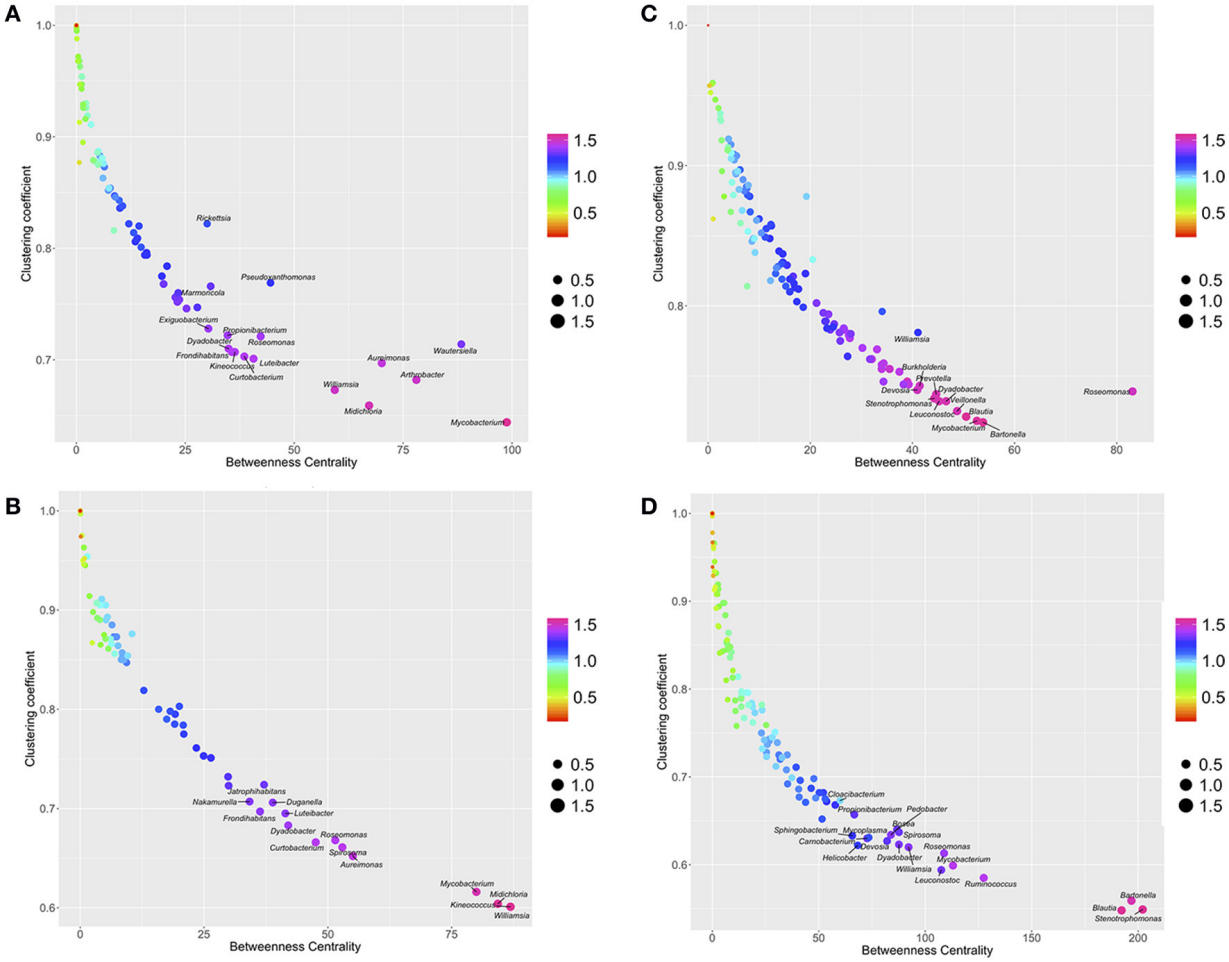
The relationships between Betweenness Centrality (BNC), Clustering coefficient (CC) and PageRank (PR) in the networks of co-occurring bacteria in ticks-forest **(A)**, voles-forest **(B)**, ticks-ecotone **(C)**, and voles-ecotone **(D)**. Each plot is colored and sized according to the values of PR and placed in the intersection of values of BNC and CC. Only most prominent genera of bacteria are indicated (complete illustrations with the labels for all the bacteria are provided in Figure S5).

The plot of the distribution of BNC and CC (Figure 2) displays the dominant presence of some bacteria in the networks: the genera located in the bottom-right of the plots have the lowest rates of co-occurrence with other genera (low CC) and a central role in the topology of the network (high BNC). The meaning of these charts is that bacteria that tend to be central in the network, have the lowest rates of co-occurrence with other genera. The bacteria occupying prominent positions are different for ticks and voles, either in ecotone or forest. Six bacterial genera (*Aureimonas, Wautersiella, Arthrobacter, Williamsia, Midichloria*, and *Mycobacterium*) have the most central positions in the network of ticks-forest (Figure 2A). Three of these bacteria (*Midichloria, Mycobacterium*, and *Williamsia*) together with the genus *Keinococcus* have the same traits in the network ticks-ecotone (Figure 2B). *Bartonella, Mycobacterium, Roseomonas, Blautia* and *Stenotrophomonas* have central positions in the networks of voles (Figures 2C,D). Figure S7 includes these charts with the complete set of labels for every detected bacterial genus.

As for the prevalence of bacterial genera, a low phylogenetic signal was obtained for BNC, CC, and PR (Table 1). These results point to a lack of correlation between bacterial lineages in the phylogenetic tree and their relative importance in the networks: closely related genera do not tend to occupy simultaneously prominent positions in the network of co-occurring microorganisms.

**Table 1:** Values of Pagel's λ as a measure of the phylogenetic signal of the centrality indexes of the networks for ticks and voles in forest and ecotone.

**Networks**	**Pagel's** λ^**^		

	BNC^*^	PR^*^	CC^*^
Ticks forest	0.119	0.460	0.272
Ticks ecotone	0.220	0.324	0.255
Voles forest	0.029	0.098	0.089
Voles ecotone	6E-05	5E-05	7E-05

* *BNC, Betweenness Centrality; PR, PageRank; CC, Clustering coefficient*.

** *A λ near 0 indicates very little phylogenetic signal in the data given the tree of bacteria and a λ near 1 indicates relatively more phylogenetic signal in the data*.

### Host specificity of bacteria in ticks and voles

**Figure 3:**
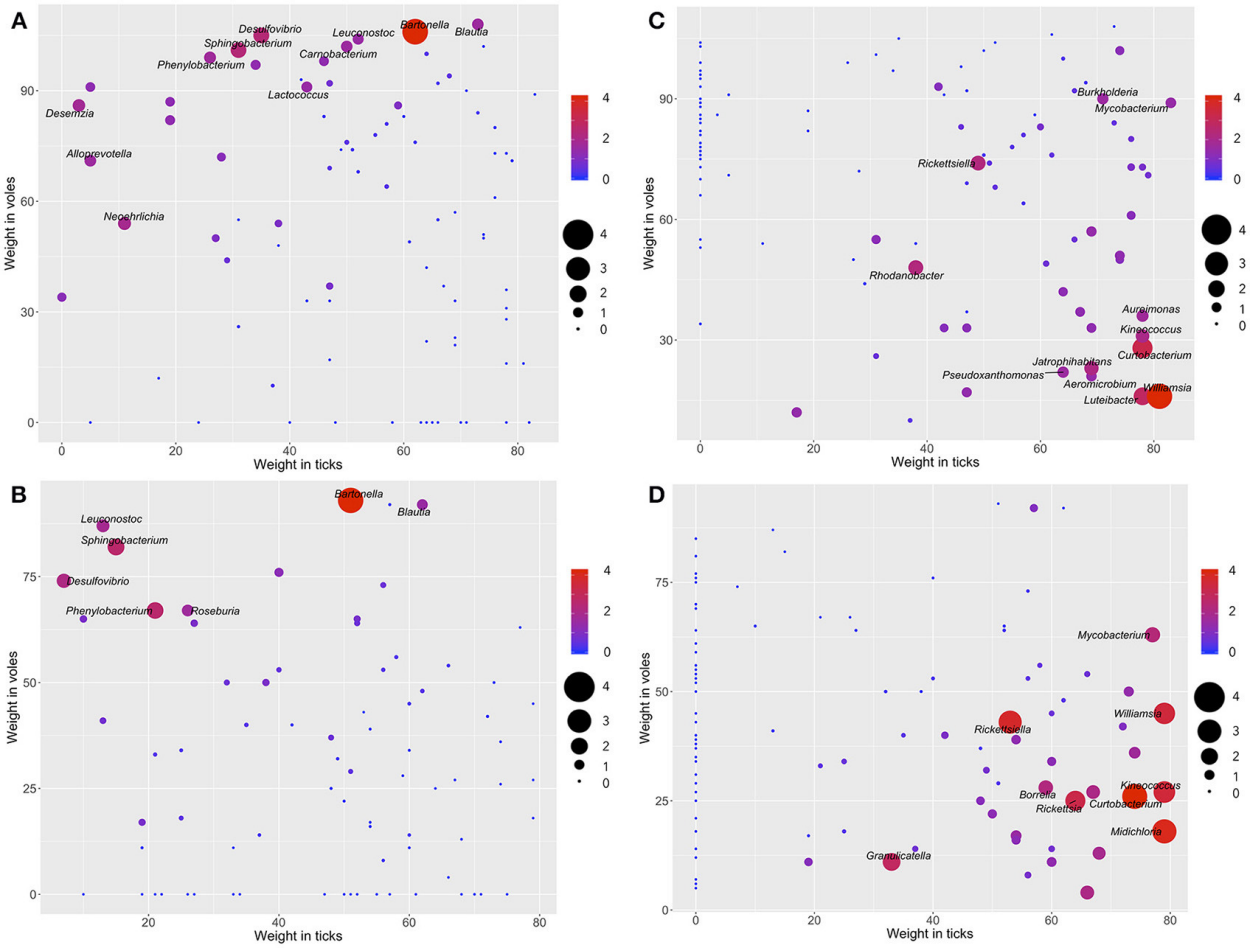
The log-ratio of the number of reads of each genus of bacteria (voles/ticks, **A,B**; ticks/voles, **C,D**) plotted along the range of weights of each genus of the network of bacteria in the biotope forest **(A,C)** and the biotope ecotone **(B,D)**. Only the labels of bacteria with higher log-ratio values are included. The color and the size of each dot and the size of the label of the genus are proportional to the log-ratio between the number of reads in voles and ticks. Genera located in the top and top-left of the charts **(A,B)**, with warm colors and large size are bacteria hypothesized to be specific of the voles. Genera located in the right and bottom portions of the charts **(C,D)**, with warm colors and large size, are bacteria hypothesized to be specific of the ticks (complete illustrations with the labels for all the bacteria are provided in Figure S6).

We assessed whether some of the detected bacteria could be unambiguously associated to a specific carrier (ticks or voles) or not. The microorganisms located on top of the chart in Figure 3 have high values of the ratio of reads and thus are defined as mainly associated to voles, and sporadically found in ticks. These include, for the forest, the genera *Bartonella, Desulfovibrio, Sphingobacterium, Neoehrlichia, Desemzia, Phenylbacterium, Alloprevotella, Blautia, Carnobacterium*, and *Leuconostoc* (Figure 3A); and for the ecotone the genera *Bartonella, Sphingobacterium, Phenylbacterium*, and *Desulfovibrio* (Figure 3B). Figures 3C,D display the same assessment for ticks in forest and ecotone, respectively. The genera *Williamsia, Curtobacterium, Luteibacter, Rhodanobacter, Rickettsiella, Jatrophihabitans, Kineococcus, Aureimonas, Pseudoxanthomonas, Burkholderia, Mycobacterium, Aeromicrobium*, are strongly associated to ticks in the forest (Figure 3C). In the ecotone, the genera *Rickettsia, Rickettsiella, Borrelia, Midichloria, Mycobacterium, Williamsia, Curtobacterium*, and *Granulicatella*, were detected as mainly associated to ticks (Figure 3D). See also the complete information in Figure S8.

### Bacterial similarity between ticks and voles

**Figure 4:**
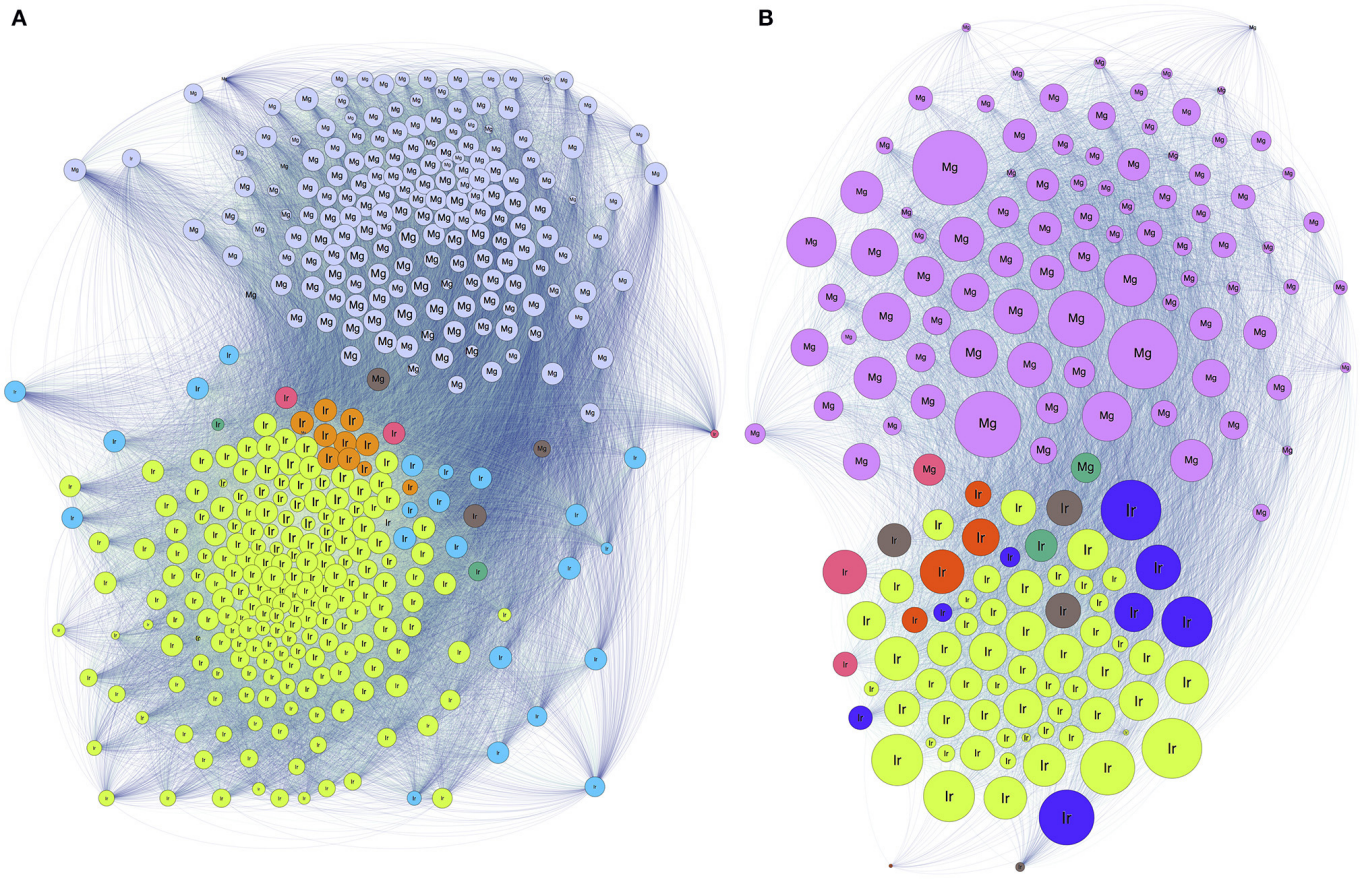
The network structure of the relationships among individual ticks and voles regarding the shared genera of bacteria in forest **(A)** and ecotone **(B)**. Colors and numbers represent different communities (as included in Figure 5). Each circle represents an individual tick (Ir) or a vole (Mg) and the links between them represent relationships. The size of the node is proportional to its BNC, and the size of the label is proportional to its PR.

**Figure 5:**
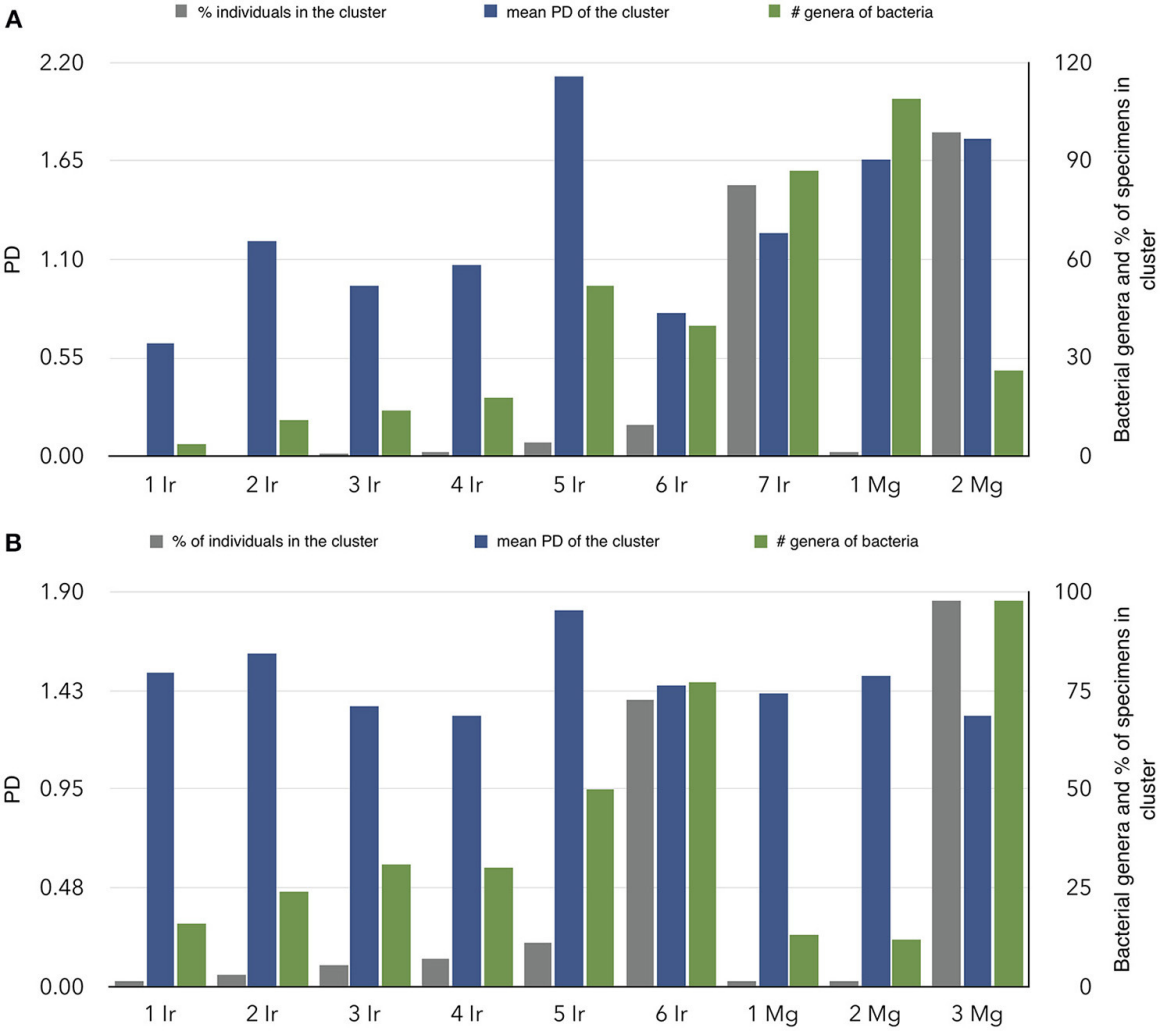
Characteristics of the communities of ticks-voles regarding the relationships of their microbiomes in forest **(A)** and ecotone **(B)**. The chart summarizes the findings of Figure 4, separately for both ticks and voles, using a clustering algorithm that detects the similarity of the bacterial fauna between pairs of individuals (ticks and/or voles). On the X-axis, communities detected in ticks are labeled as Ir and a consecutive number, and communities of voles as Mg and its number. Communities are sorted according to the percent of individuals (ticks or voles) in each cluster. For each community, vertical bars indicate the percentage of ticks or voles in that community, the number of bacterial genera in the community and the mean phylogenetic distance of the community.

We addressed the relationships between individual ticks and voles capturing the patterns of similarity among carriers according to the bacteria detected in individual ticks and voles. Results confirm the high dissimilarity of bacterial fauna between ticks and voles, in each biotope, since both carriers are split in deeply unrelated communities (Figure 4). The diversity of communities is slightly different in the forest (7 communities for ticks, 2 for voles) and in the ecotone (6 for ticks, 3 for voles). The Figure 5 shows the values of phylogenetic diversity, bacterial genera richness, and number of individuals (ticks or voles) included in each community. In the forest, one single community (7 Ir) includes the highest number of ticks (more than 80%) circulating 87 genera of bacteria, with a PD of only 1.25. The remaining six communities of ticks include 18% of individuals, carrying variable number of genera of bacteria (from 4 to 52). However, some of these communities represented by a few ticks circulate a higher PD than the largest one, with an accumulated PD of 6.78. In the forest, the 18% of ticks circulate the highest phylogenetic diversity of bacteria. In ecotone, the biggest community includes 77 genera of bacteria and 72% of ticks, accounting for a PD of 1.44. The remaining combined communities account for 28% of the ticks, carrying a variable number of genera of bacteria (16 to 50) and a combined PD of 7.58. These results clearly demonstrate that approximately 20% of ticks carry seven times more PD than the remaining 80% of ticks, results being consistent for both forest and ecotone. The microbiome of voles is more homogeneous as only 2–3 communities were detected in both forest-ecotone, respectively. Therefore, the microbiome of voles is more similar among individuals than that of ticks.

## Discussion

Early reports on arthropod microbiomes have been driven by the advances of the high throughput sequencing platforms, accessing large amounts of data and appreciating their high complexity (Hayes and Burgdorfer, 1982; Noda et al., 1997; Sacchi et al., 2004; Scoles, 2004). Most studies on ticks were directed to capture the variability of tick microbiomes while minimizing biases due to DNA preparation and bioinformatics analyses (Goodrich et al., 2014; Galan et al., 2016). These studies gave impressive insights on the origin and evolution of the bacteria detected in ticks (Narasimhan and Fikrig, 2015).

We specifically aimed to propose a common comparative framework for analysis of bacterial microbiomes, pinpointing the ecological relationships of co-occurring bacteria, using as proof of concept the data recorded for a common tick in Europe and one of its main hosts. With a few exceptions (Ruan et al., 2006; Chaffron et al., 2010; Freilich et al., 2010; Barberán et al., 2012), network analysis has not been applied to detect co-occurrence patterns in complex microbial communities or has only been used as visual depiction to these patterns. We therefore, outlined procedures facilitating a strict ecological interpretation of the microbiomes of organisms. The networks allow (i) to cluster the bacteria according to their co-occurrences, describing associations within the microbiome, and (ii) to rank the importance of each microorganism within the microbiome. Additional procedures allow the capture of the phylogenetic diversity circulated by either ticks or voles.

### Interactive effect of biotope and hosts on the microbiome of ticks

While this study is specifically aimed to develop a common framework for comparison of the microbiome of organisms retaining the ecological meaning of associations, our approach allowed demonstrating that the microbiome of the tick *I. ricinus* is only slightly related to the microbiome of one of its main hosts, *M. glareolus*. Therefore, the microorganisms present in one of the main hosts for the tick is not an indicator of the microbiome of the tick. This strongly suggests that crude pair-wise comparison of microbiomes of arthropod vectors and their hosts lack ecological meaning. In agreement with our results, bacterial communities of *Dermacentor variabilis* and *I. scapularis* were significantly different to that of the blood of their shared host *Peromyscus leucopus* (Rynkiewicz et al., 2015). In contrast, Swei and Kwan (2017) showed that the tick microbiome of *I. pacificus* was strongly influenced by host blood meal. Another clear-cut result of this study is that bacterial assemblages of both ticks and voles are influenced by the biotope: microbiomes of voles or ticks collected in different biotopes share a very small proportion of bacteria genera. This has been also demonstrated in a recent study by Zolnik et al. (2018) where the environment was found to play a significant role in shaping the internal microbiome of *I. scapularis*.

The influence of the biotope on tick and vole microbiomes could be due to different factors. Microbiome composition could be largely influenced by the interactions with the environment, given that bacteria in ticks could be acquired directly from the soil or from plants (Zolnik et al., 2016). As soil and vegetation would be different in both biotopes, bacterial assemblages related to soil and plants would also be, influencing tick microbiome. Another important process of bacterial acquisition by ticks and voles would result from the life cycle of the ticks, with three developmental stages, each feeding on a different host. Since the host composition of both biotopes is expected to be different, the bacterial accumulation results from the trans-stadial (from one tick stage to the next) and/or trans-ovarian (from the tick female to the eggs) passage of most bacteria, which result from a blood meal on different vertebrates. The framework presented in this study provides a methodology that could be widely applied to capture the patterns of microbial interactions in the tick.

### Structure of the microbiomes in ticks and voles

An interesting result of this study suggests that there are not bacteria indispensable for the co-occurrence of other genera. The networks lack large hubs, as it could be expected from co-occurrence patterns in which the presence of a microorganism is pivotal for the occurrence of the others. These networks have a structure called “small world,” an effect originally observed in a social study (Milgram, 1967), and subsequently shown in neural networks (Watts and Strogatz, 1998). This scale-free property strongly correlates with network robustness, the lack of hierarchy allowing for a fault tolerant behavior. Therefore, networks resulting from our analyses are very resilient: the removal of one node of the network would impact only slightly the connectivity of the others, which suggest the lack of trophic dependence or competition between co-occurring genera. This is an important conclusion that demonstrates that most central bacteria (i.e., those with high prominence in the network) have low phylogenetic affinities. This finding could be interpreted as a way to circumvent the co-occurrence of closely related bacteria, probably with similar metabolic needs, in either ticks or voles. This finding fuels the interest to understand the molecular relationships and the synergetic or antagonist mechanisms that microorganisms may express in the tick.

### Specificity of the microbiomes of voles and ticks

The networks provided extra information about the specificity of co-occurring bacteria. The context is to pinpoint if a microorganism is specific of the vertebrate, and circumstantially circulated by the tick, or if it is specific of the tick, therefore occurring inconsistently in the vole. We demonstrated that i.e., *Bartonella* is clearly associated to voles. Read numbers of *Bartonella* are 16,857 and 17,478 times higher in voles than in ticks, in forest and in ecotone, respectively. Therefore, its presence in ticks may to be considered as a casual finding and its transmission by ticks as an accidental event, as suggested by Telford and Wormser (2010). Concerning *Borrelia*, this bacterium circulates among different vertebrates and ticks (Cutler et al., 2017) and the issue of its original host still persists (Gatzmann et al., 2015). *Borrelia* is a microorganism that depends on both vertebrates and ticks to circulate (Margos et al., 2017). *Borrelia* appeared 29 and 36 more times in ticks than in voles, in forest and in ecotone, respectively. However, it is important to stress that the spleen is not the best organ for detection of *Borrelia* in vertebrates. Results for both *Rickettsia* and *Ricketsiella*, which are clearly associated to ticks, support the proposed framework.

In the system we studied, microorganisms circulate together in “packets,” resulting in redundant networks highly tolerant to collapse. Noteworthy, ticks carry a larger phylogenetic diversity of bacteria than voles. This point suggests that interactions among bacteria, as well as between bacteria and carriers, have different mechanisms of regulation. The microbiome in ticks could be directly related to the host community of each habitat, a point not tested in this study, which warrants further research. Alternatively, it could be hypothesized that ticks impose higher selection of microorganisms than do the voles, allowing only some combinations of particular bacteria, and thus a variety of possible re-arrangements. Most voles, however, have a similar microbiome composition. The results clearly pointed out that a few specimens of ticks carried the highest PD in both forest and ecotone, while the large majority of ticks (about 80%) circulated lower values of PD than the resulting sum of the rest 20%. These data also support the hypothesis of a richer bacterial community in ticks gained by the feeding on a diverse array of hosts, resulting in very diverse assemblages of bacteria, that follow the classic Pareto distribution of 20/80 (i.e., the 20% of ticks carry the 80% of PD) even if the number of bacterial genera is lower.

This study provided a reproducible and coherent framework for detecting the ecological relationships among the bacteria detected in an arthropod and one of its main vertebrate hosts. It has deep roots on the graph theory applied to inter-organism relationships. The approach has applications to many diverse circumstances in which an explicit comparison of interacting organisms is necessary. With the increasing availability of high resolution sequencing methods, methods to summarize the relationships between microorganisms are a cardinal part of the analysis. It is however necessary to analyse the synergistic or antagonistic relationships between co-occurring bacteria, as the next step in understanding their molecular relationships and their effects on tick-transmitted pathogens.

## Author contributions

AE-P, J-FC, and AC-C conceived the work, wrote the manuscript. AE-P, J-FC, TP, and AC-C performed data analysis. All authors revised the manuscript.

### Conflict of interest statement

The authors declare that the research was conducted in the absence of any commercial or financial relationships that could be construed as a potential conflict of interest.

## Supplementary material

Figure S1 The phylogenetic tree of all the genera of bacteria detected in this study, as obtained from the data in Tables S1, S2.

Figure S2 The figure displays a guide to the application of network analysis to microbiome studies. Firstly, co-occurrence (i.e., the number of times a genus co-occurs with other genera of bacteria) of microbial taxa among the samples is calculated. The co-occurrence table is used as input for network analysis software **(A)**. The co-occurrence table is used to calculate important indexes including the Degree, Centrality and Modularity of the network. The software provides a plain and basic visual depiction of the network **(B)** that can be enhanced by coloring the communities of nodes and their links, and resizing each circle representing the microorganism by a value, in this case the Centrality **(C)**. Phylogenetic tree of bacterial genera is built using evolutionary distances calculated from pairwise comparisons of full length 16S DNA sequences available for bacteria species within each genus **(D)**. This tree can be used to calculate the phylogenetic distance among the taxonomic ranks of bacteria, track the phylogenetic signal (using Pagel's λ) of the indexes of the networks and evaluate the phylogenetic composition of the communities of co-occurring bacteria **(D)**.

Figure S3 Rarefaction curves estimated from reads obtained for voles in forest **(A)** and ecotone **(B)** and ticks in forest **(C)**, and ecotone **(D)**.

Figure S4 Frequency distribution of the number of bacterial general recorded per tick/vole in either forest or ecosystem. **(A)** ticks, forest; **(B)** voles, forest; **(C)** ticks, ecotone; **(D)** voles, ecotone.

Figure S5 Frequency distribution of the number of reads recorded per tick/vole in either forest or ecosystem. **(A)** ticks, forest; **(B)** voles, forest; **(C)** ticks, ecotone; **(D)**: voles, ecotone.

Figure S6 The networks of the genera of bacteria detected in ticks-forest **(A)**, voles-forest **(B)**, ticks-ecotone **(C)**, and voles-ecotone **(D)**. Colors mean for different communities of bacteria, i.e., bacteria that appear together with a higher affinity among them than with others. The size of each node is proportional to the Betweenness Centrality, and the size of the label is proportional to the PageRank of the node. Lines mean for co-occurrences between nodes, with the same color that the nodes of origin, its width proportional to the number of co-occurrences between two genera. The chart shows “spirals” to display the communities of the genera of the microorganisms, sorted according to its Betweenness Centrality.

Figure S7 The relationships between Betweenness Centrality (BNC), Clustering coefficient (CC) and PageRank in the networks of co-occurring bacteria in ticks-forest **(A)**, ticks-ecotone **(B)**, voles-forest **(C)**, and voles-ecotone **(D)**. Each plot is colored and sized according to the values of PageRank and placed in the intersection of values of BNC and CC. The information is the same as in Figure 2 but including labels for all the bacteria.

Figure S8 The log-ratio of the number of reads of each genus of bacteria (voles/ticks or ticks/voles) plotted along the range of weights of each genus in the four networks of co-occurring bacteria. The color and the size of each dot and the size of the label of the genus show the log-ratio between the number of reads. The information is the same as in Figure 3 but including labels for all the bacteria. **(A)** voles/ticks, forest; **(B)** voles/ticks, ecotone; **(C)** ticks/voles, forest; **(D)** ticks/voles, ecotone.

Table S1 The set of complete raw data used in this study, for each individual vole or tick, in either forest or ecotone. This is the dataset used for all calculations and to build the networks. The table includes information about the carrier (tick or vole), the sex of the carrier (M: male; F: female), the landscape (forest or ecotone), and the number of OTUs and reads obtained for each individual sample.

Table S2 16S rDNA nucleotide sequences of the genera of 126 bacteria detected in either ticks or voles, in “fasta” format, and used to build the phylogenetic tree of detected microorganisms.

Table S3 Genetic distances of the genera of bacteria detected in this study, obtained from the sequences detailed in Table S1. The accession number of each sequence in GenBank is included together with the genus of bacteria.

Table S4 Data about all the genera of bacteria (including higher taxonomic ranks, like Family and Order) detected in voles or ticks. This table includes the percentage of times that each genus appeared in a carrier (voles or ticks), and the number of reads of each genus found in either voles or ticks, in either forest or ecotone. In example, the genus *Midichloria* was detected in more than 80% of ticks but less than 1% of voles.

Table S5 General data about the indexes of the four networks generated for voles or ticks, in either forest or ecotone. Included are the genera of bacteria, and for each genus the values of Weight, Betweenness Centrality, Closeness Centrality, Eigenvector Centrality, PageRank and Clustering coefficient.
