# Supplementary figures and images for: High Throughput Sequencing and Network Analysis Disentangle the Microbial Communities of Ticks and Hosts Within and Between Ecosystems
Source: Front Cell Infect Microbiol. 2018 Jul 9;8:236. doi: 10.3389/fcimb.2018.00236 (PMC6046413; doi:10.3389/fcimb.2018.00236)

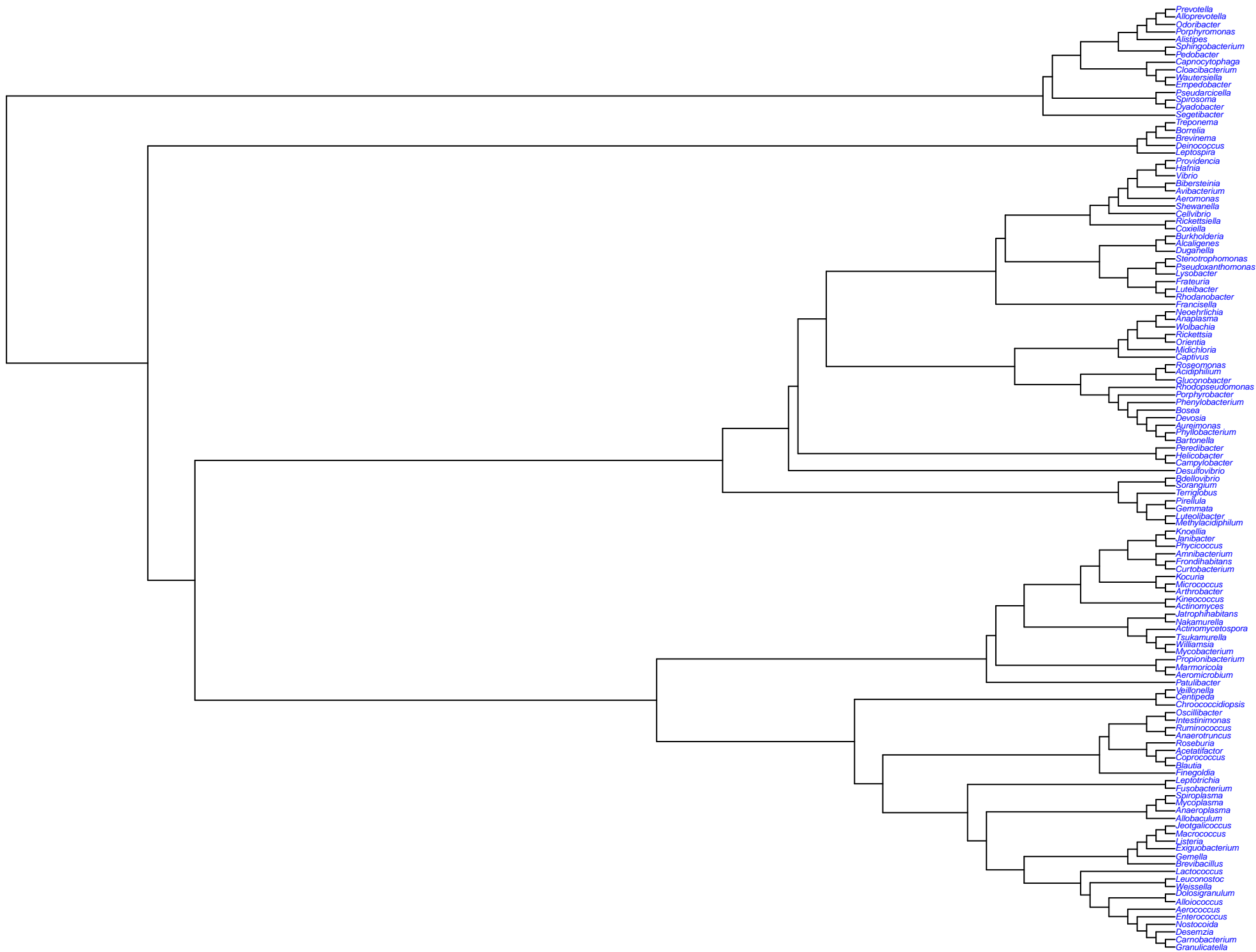

Supplement: Figure S1 — The phylogenetic tree of all the genera of bacteria detected in this study, as obtained from the data in Tables S1, S2. [file Image_1.PDF]

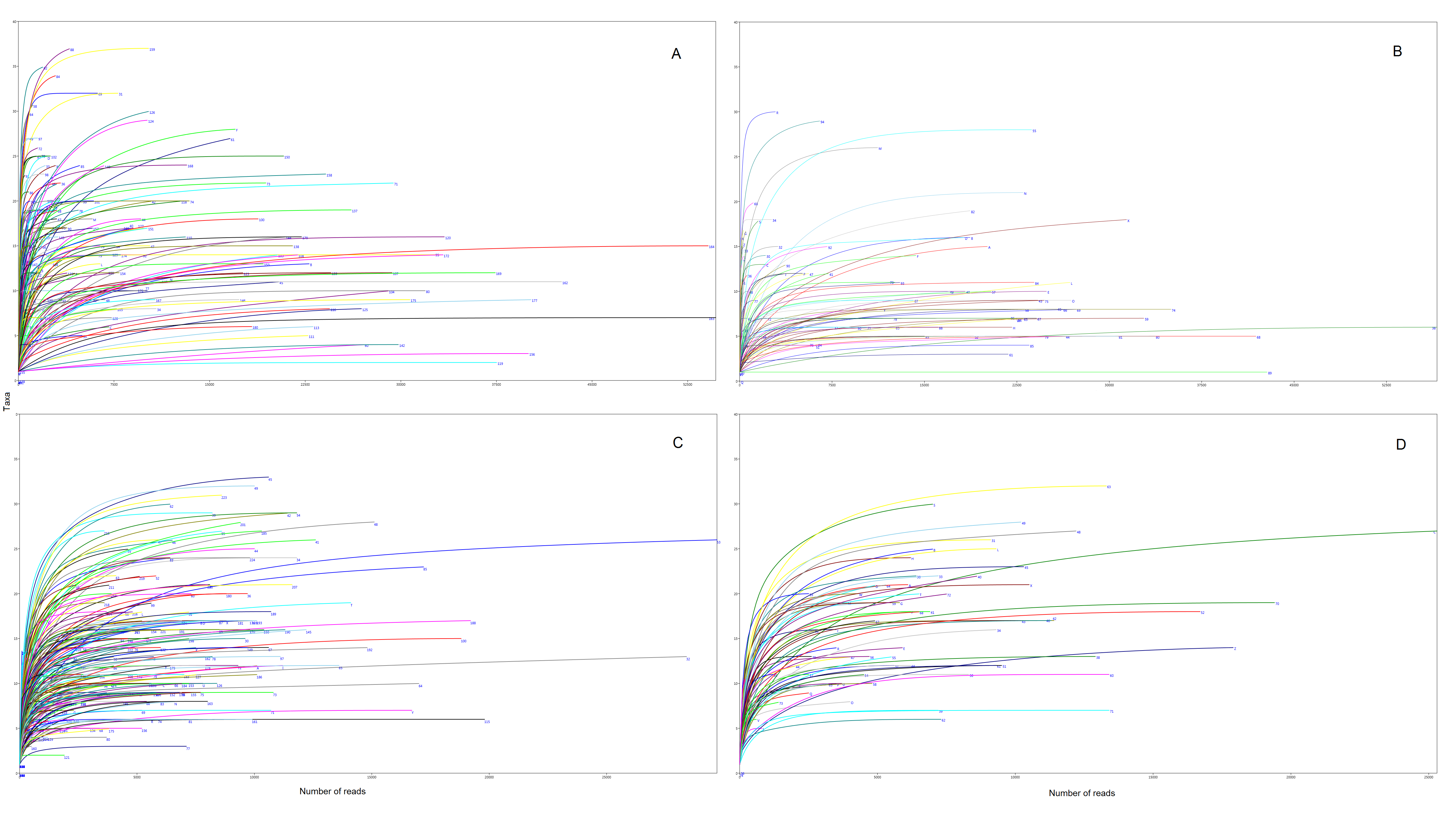

Supplement: Figure S3 — Rarefaction curves estimated from reads obtained for voles in forest (A) and ecotone (B) and ticks in forest (C), and ecotone (D). [file Image_3.TIFF]

A

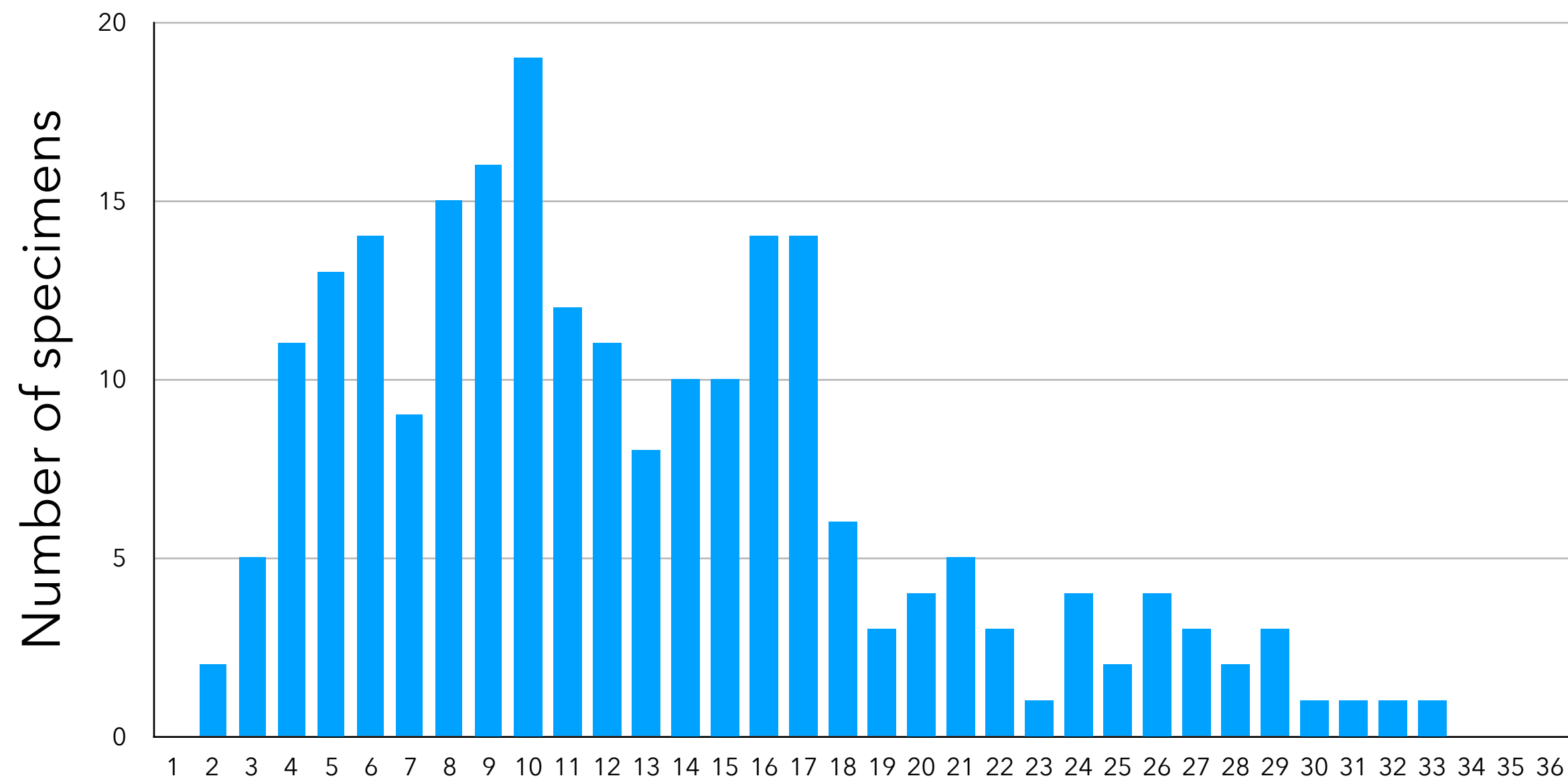

B

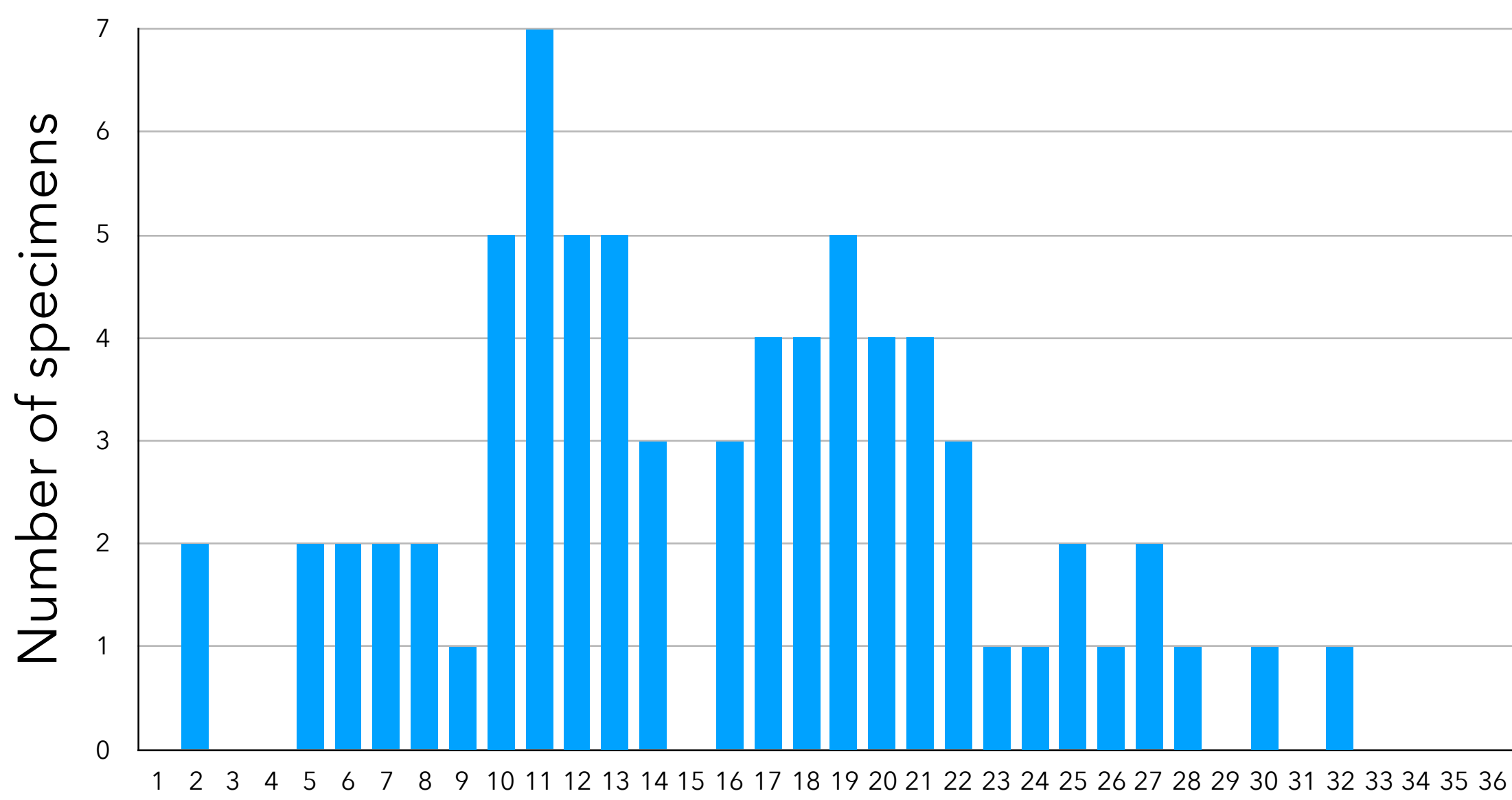

C

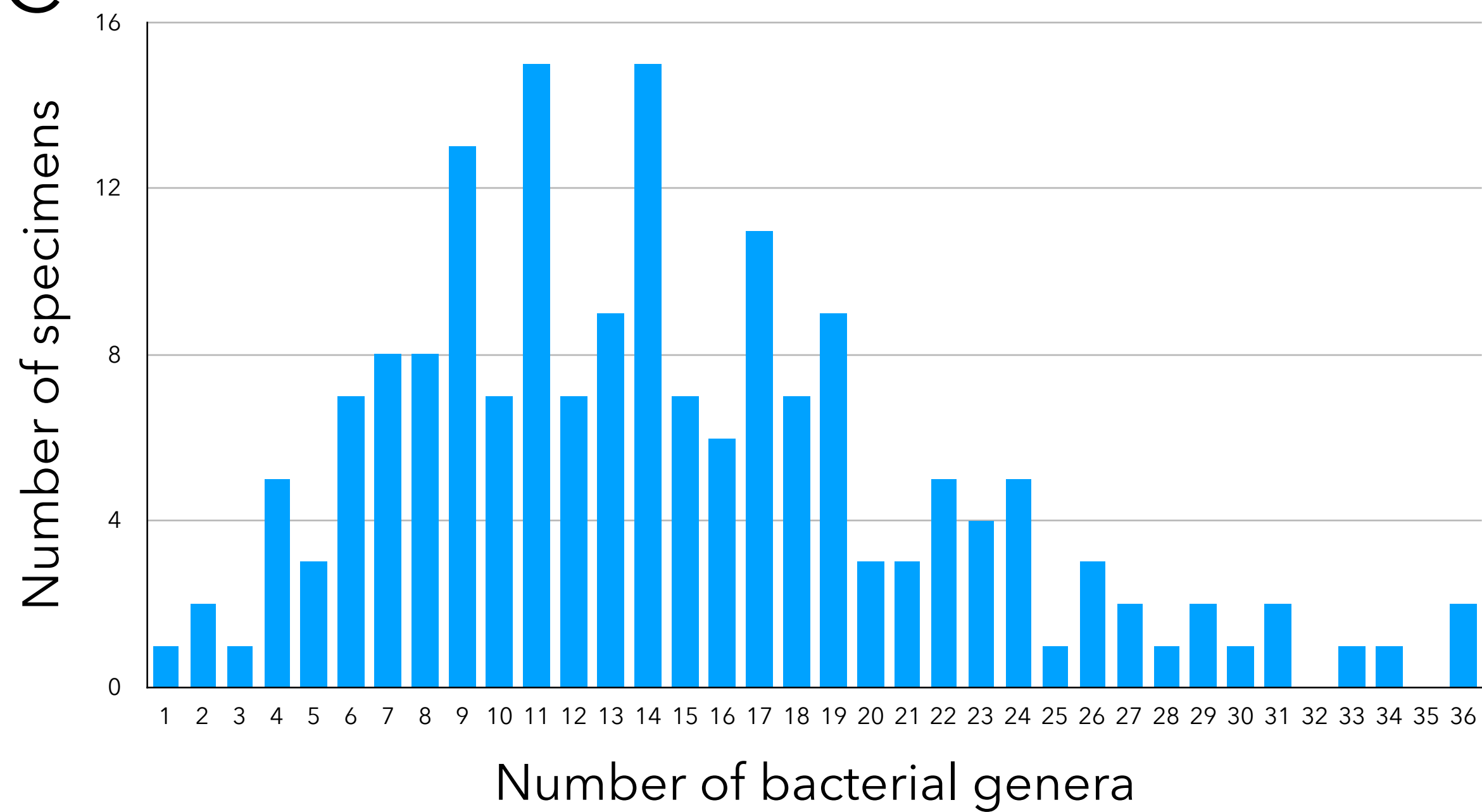

D

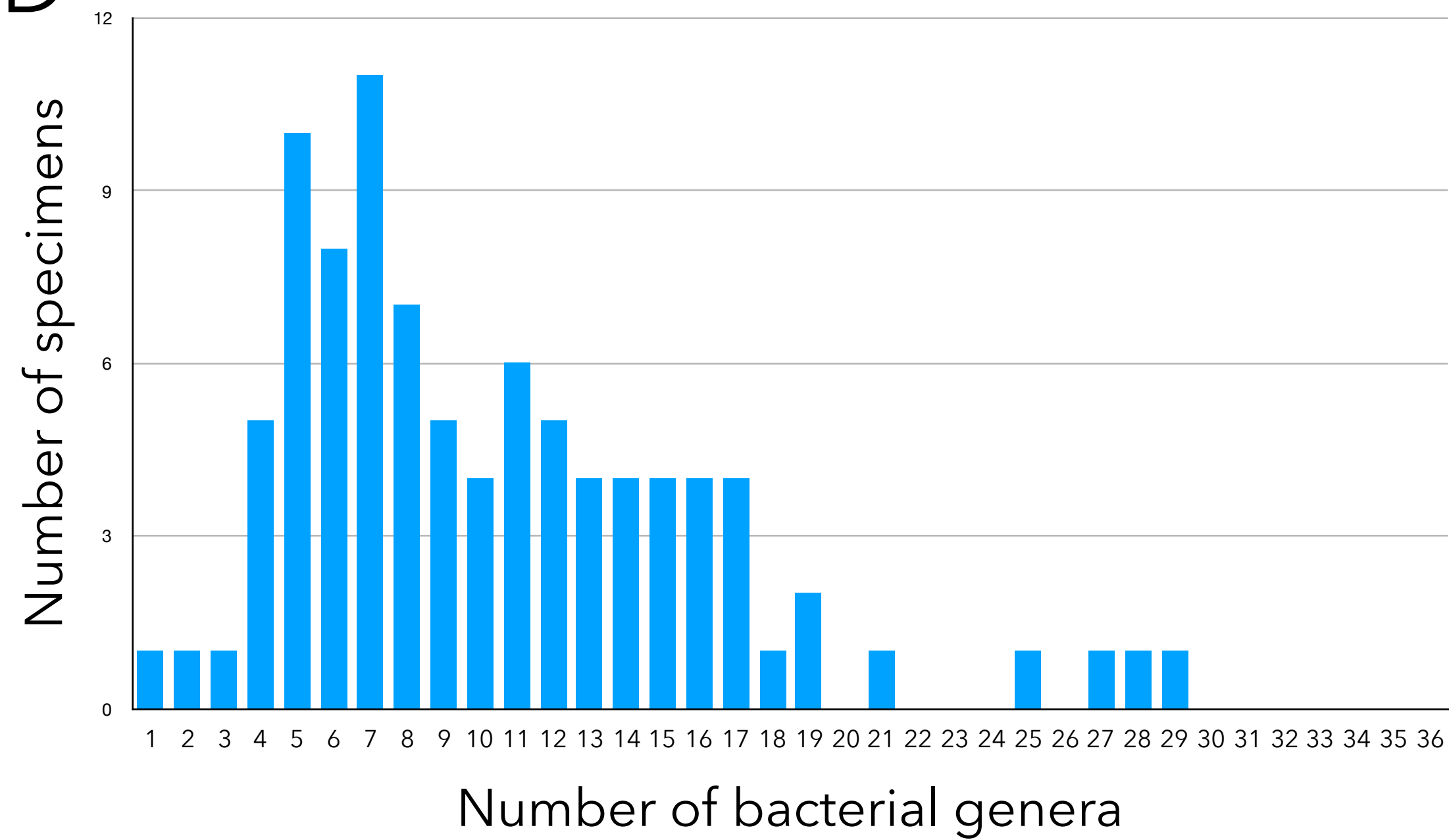

Supplement: Figure S4 — Frequency distribution of the number of bacterial general recorded per tick/vole in either forest or ecosystem. (A) ticks, forest; (B) voles, forest; (C) ticks, ecotone; (D) voles, ecotone. [file Image_4.PDF]

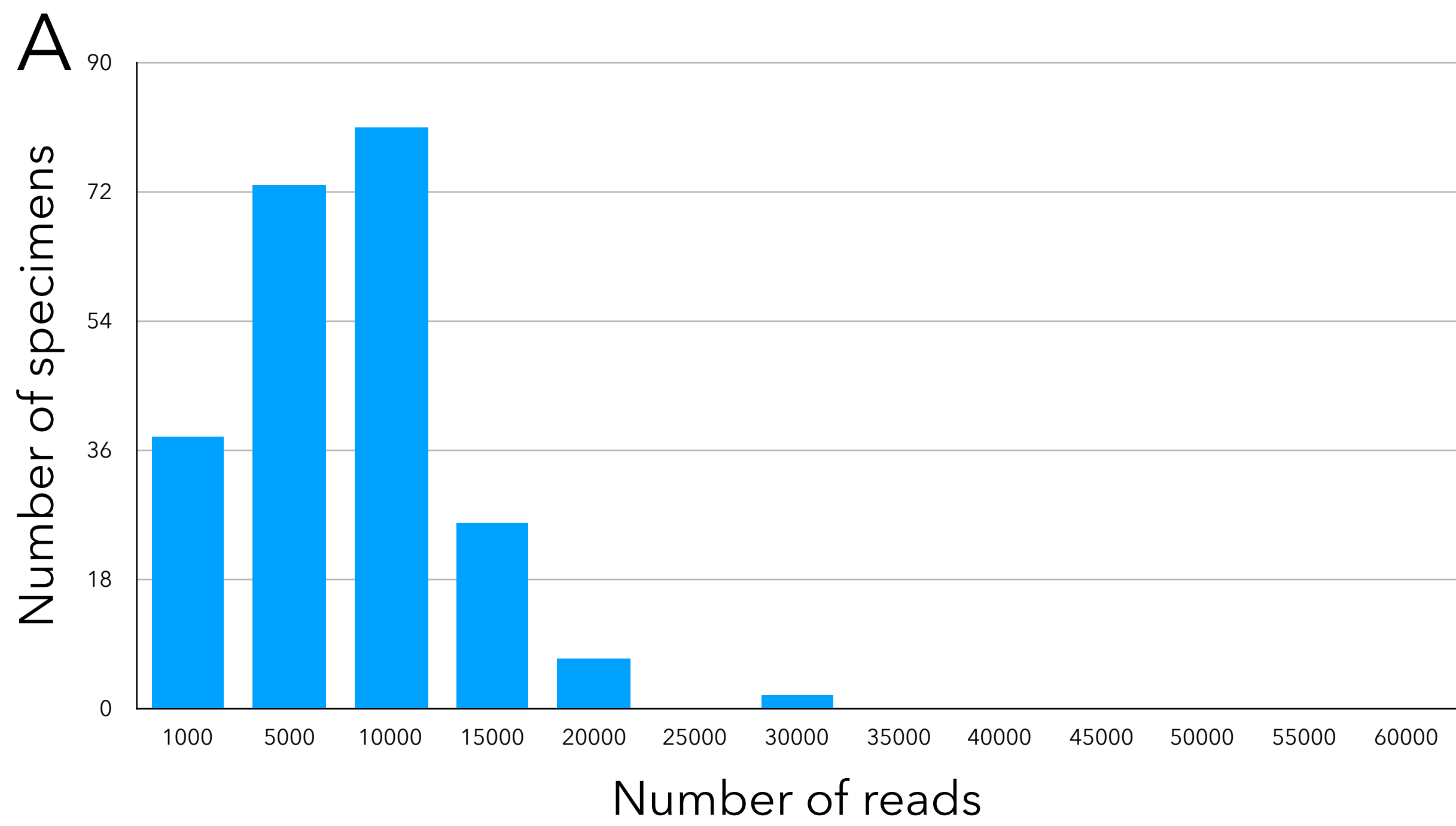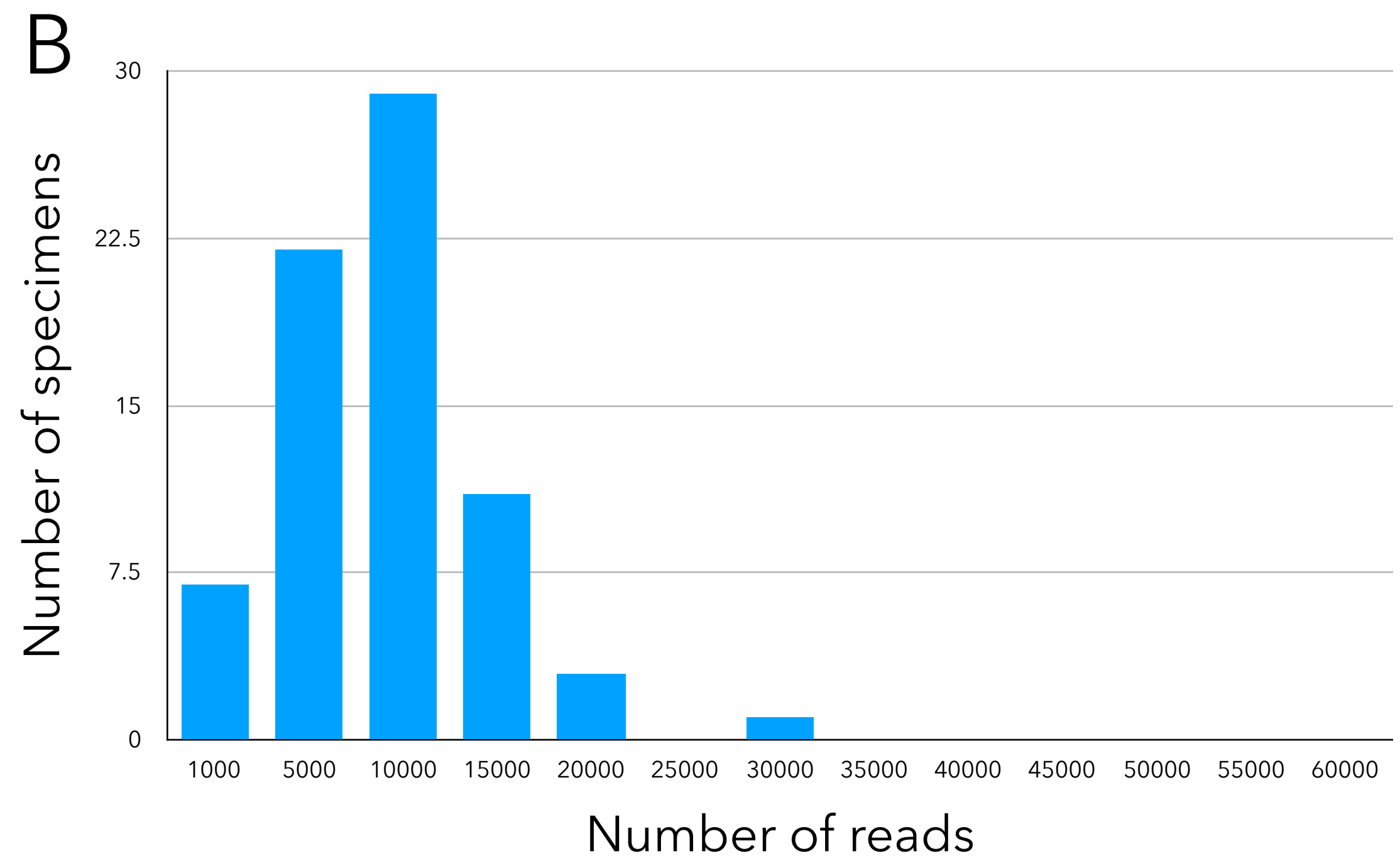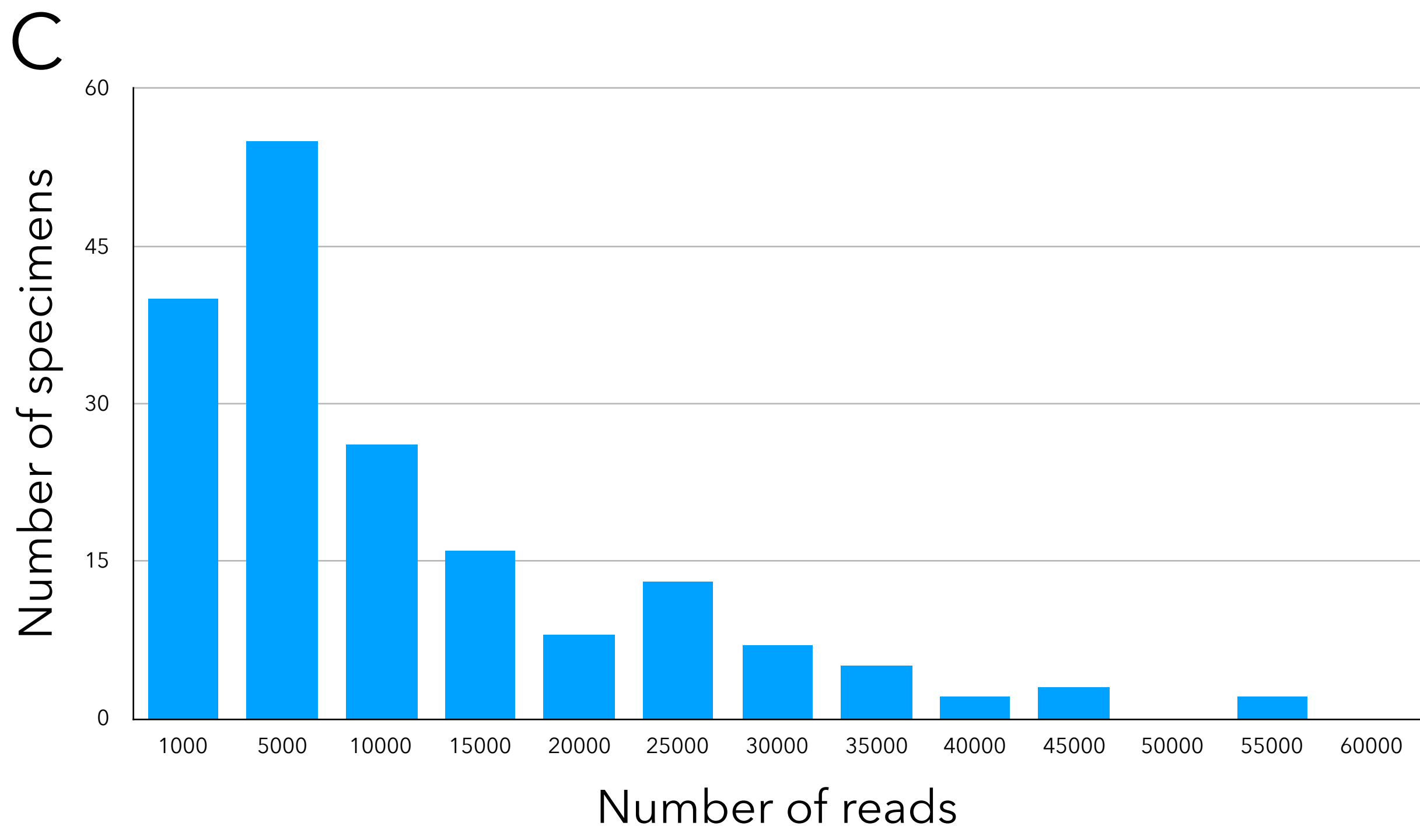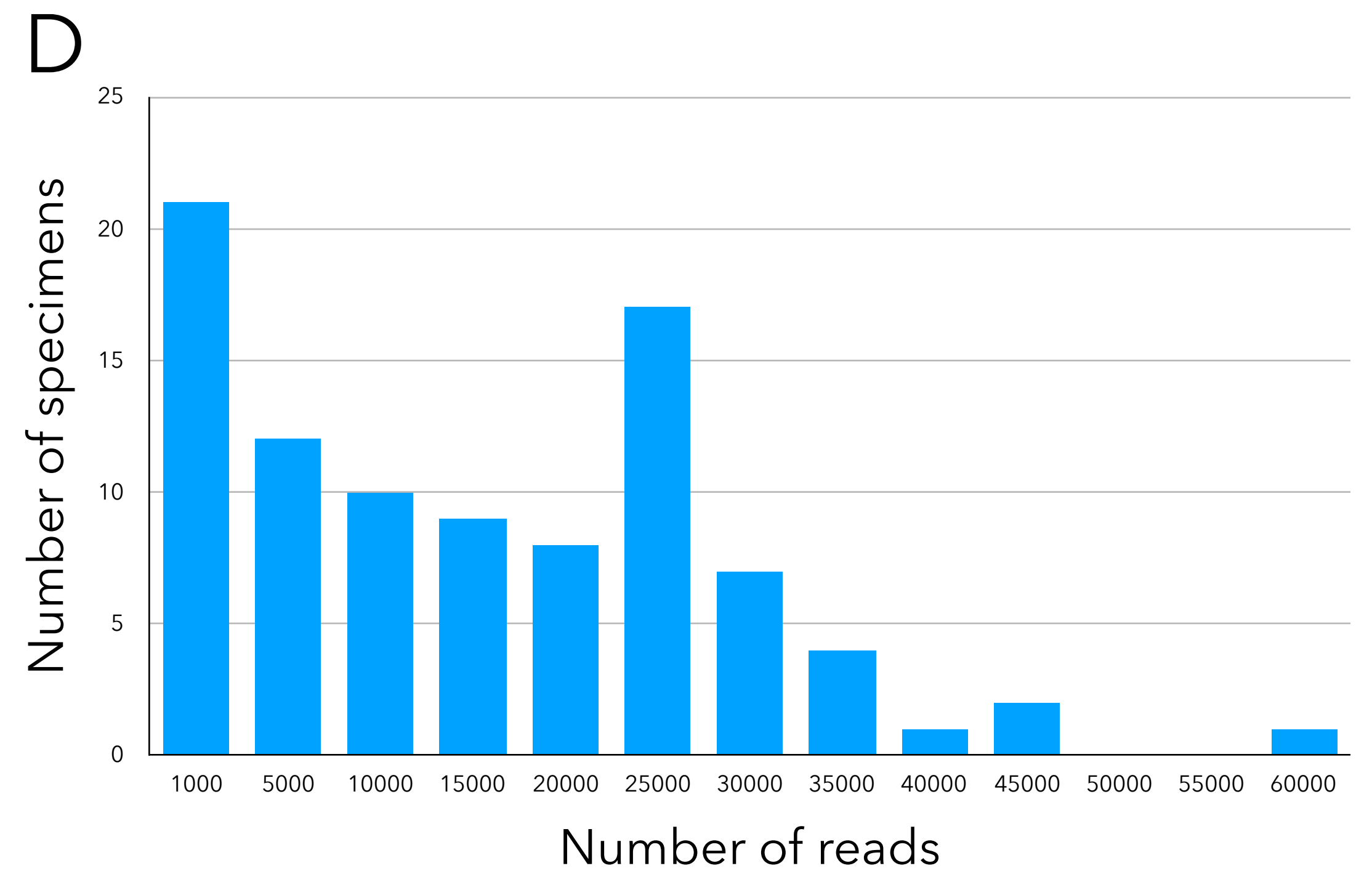

Supplement: Figure S5 — Frequency distribution of the number of reads recorded per tick/vole in either forest or ecosystem. (A) ticks, forest; (B) voles, forest; (C) ticks, ecotone; (D): voles, ecotone. [file Image_5.PDF]

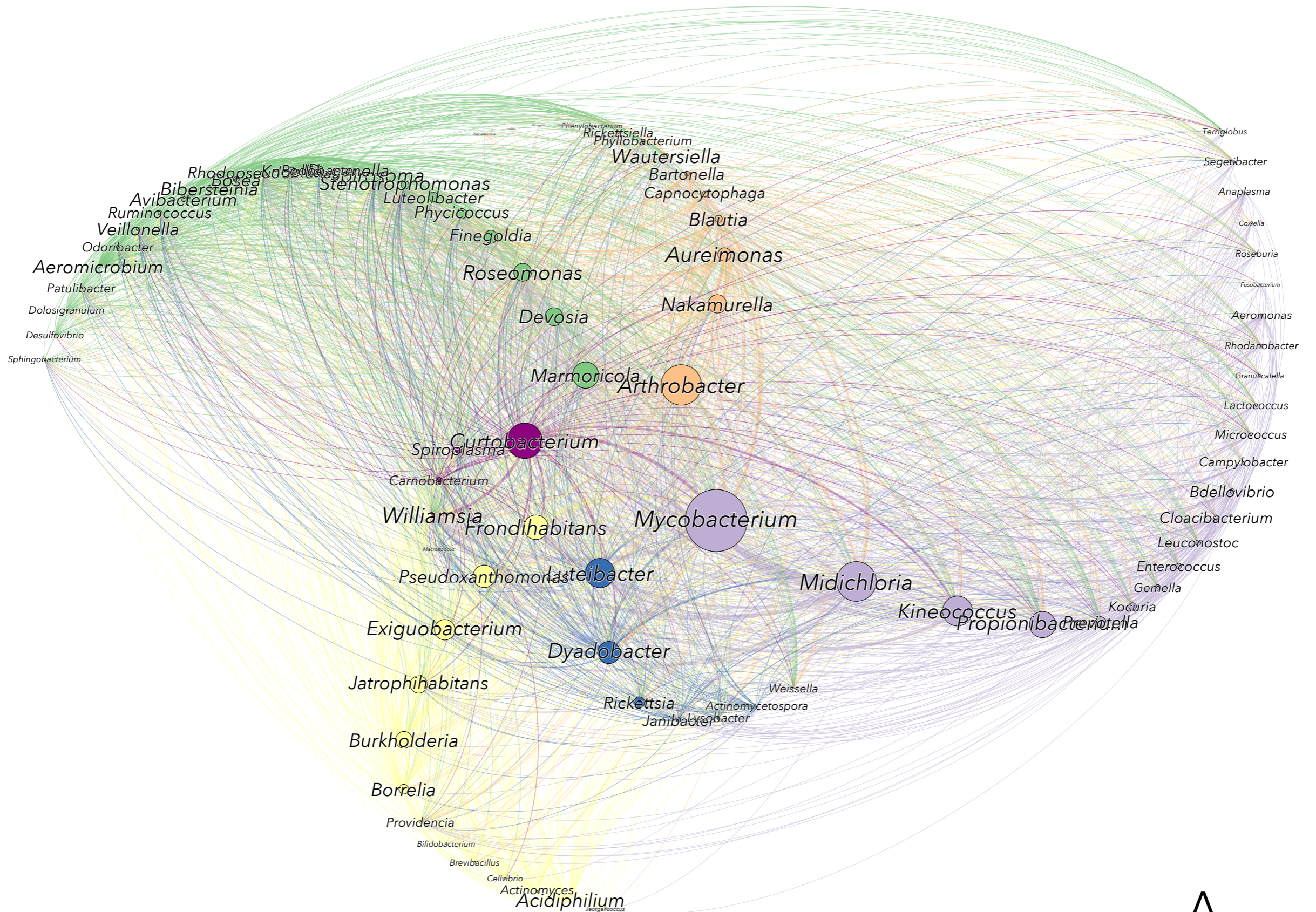

A



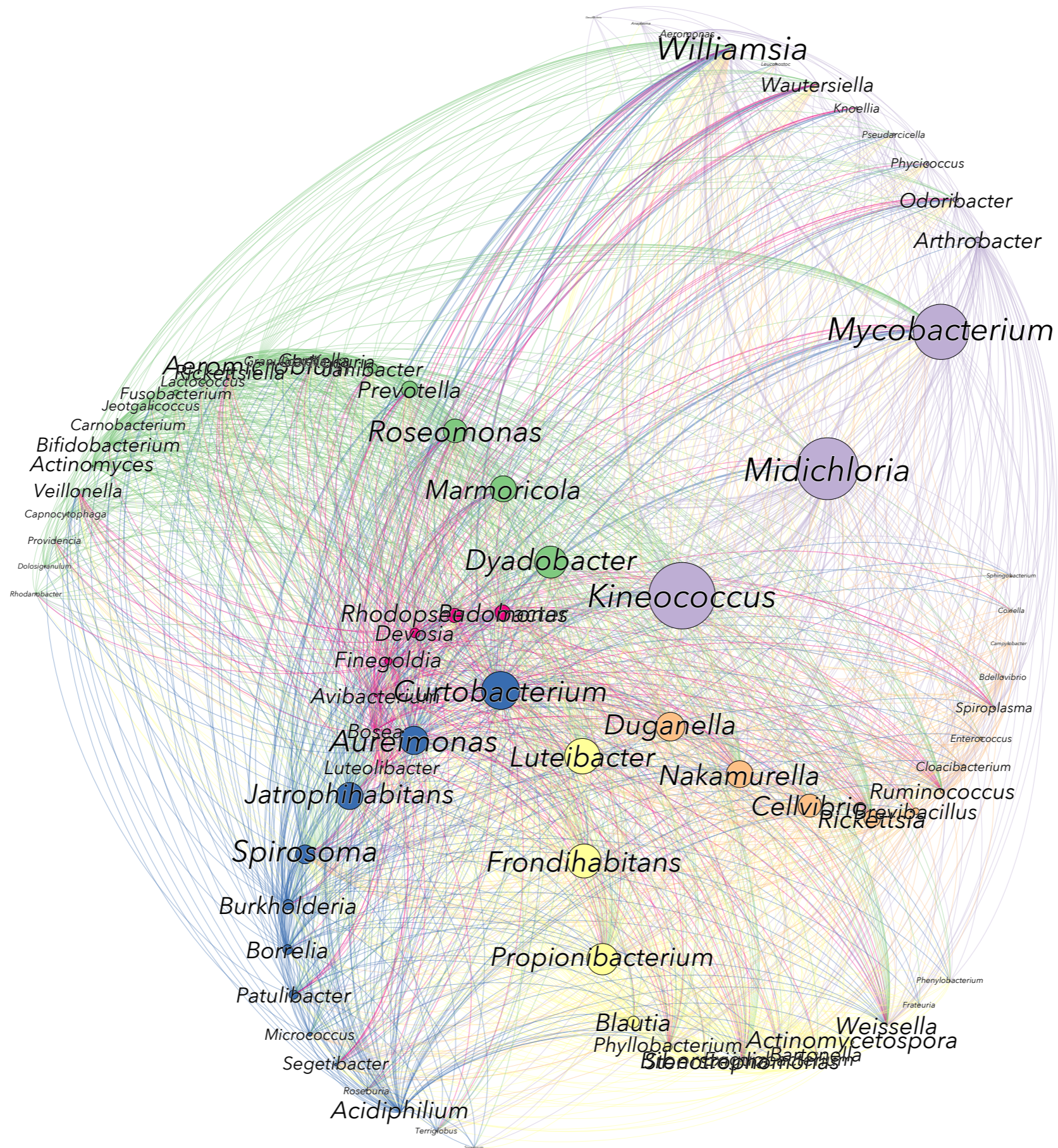

C

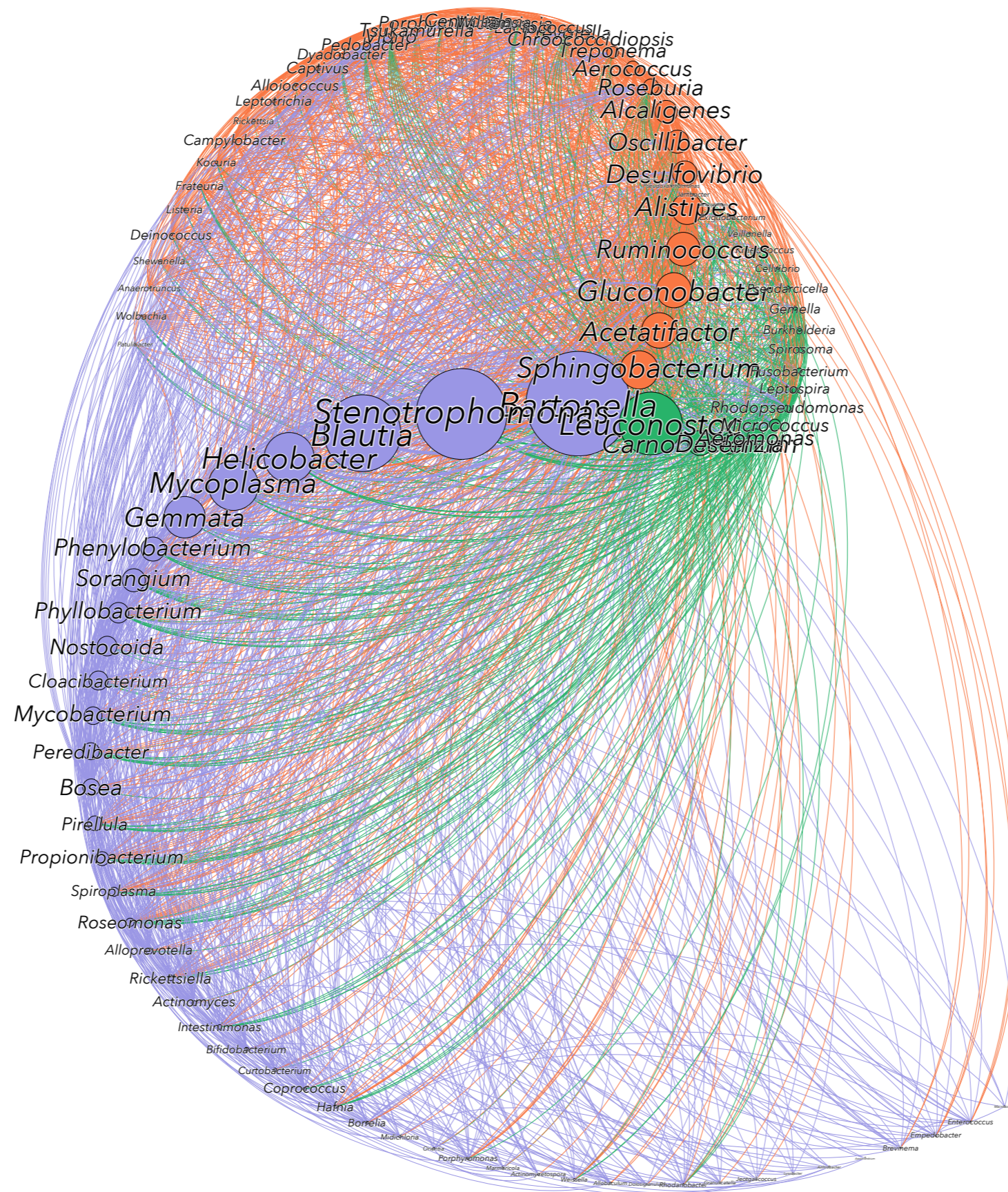

D

Supplement: Figure S6 — The networks of the genera of bacteria detected in ticks-forest (A), voles-forest (B), ticks-ecotone (C), and voles-ecotone (D). Colors mean for different communities of bacteria, i.e., bacteria that appear together with a higher affinity among them than with others. The size of each node is proportional to the Betweenness Centrality, and the size of the label is proportional to the PageRank of the node. Lines mean for co-occurrences between nodes, with the same color that the nodes of origin, its width proportional to the number of co-occurrences between two genera. The chart shows “spirals” to display the communities of the genera of the microorganisms, sorted according to its Betweenness Centrality. [file Image_6.PDF]

**A**

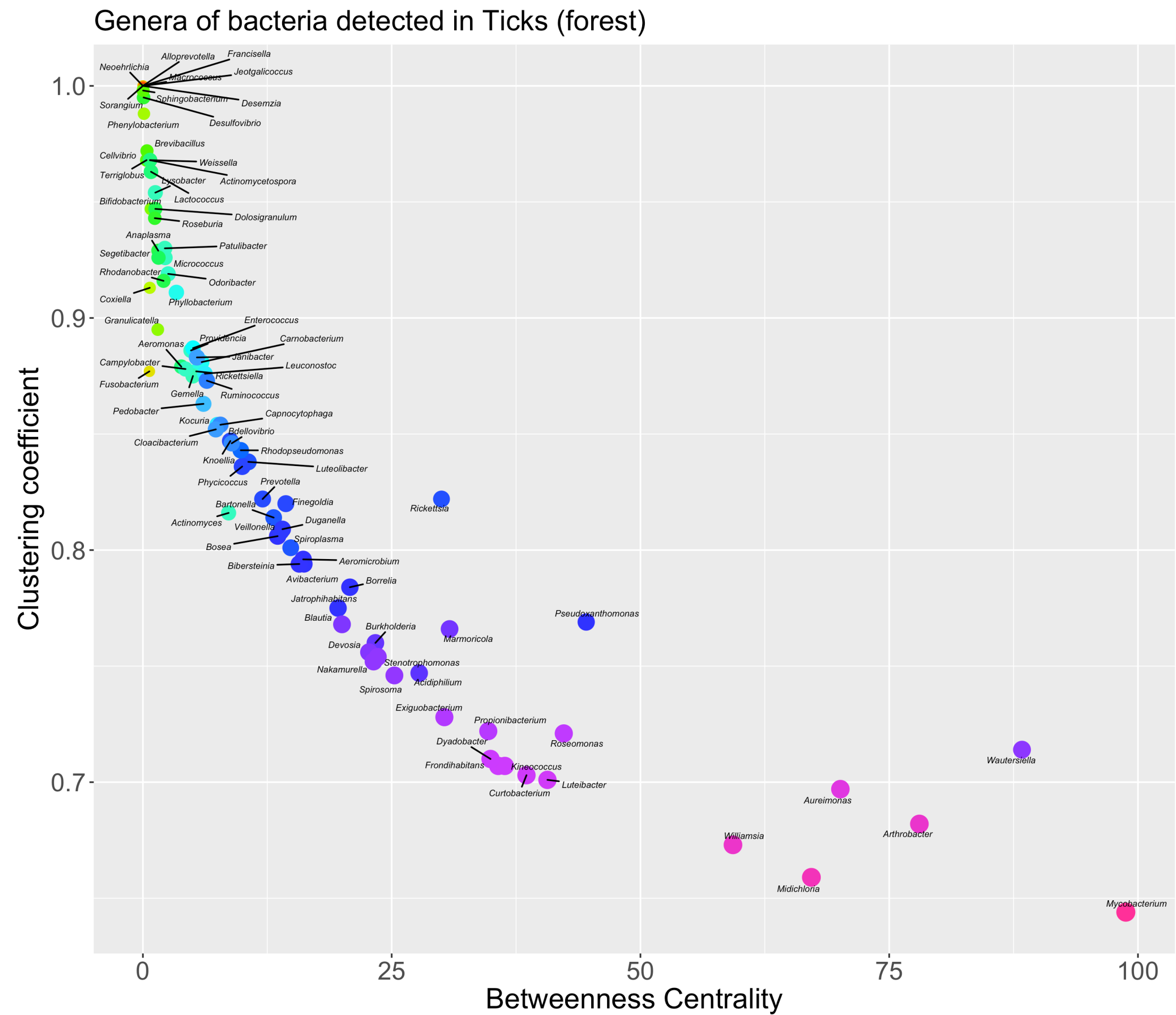

B

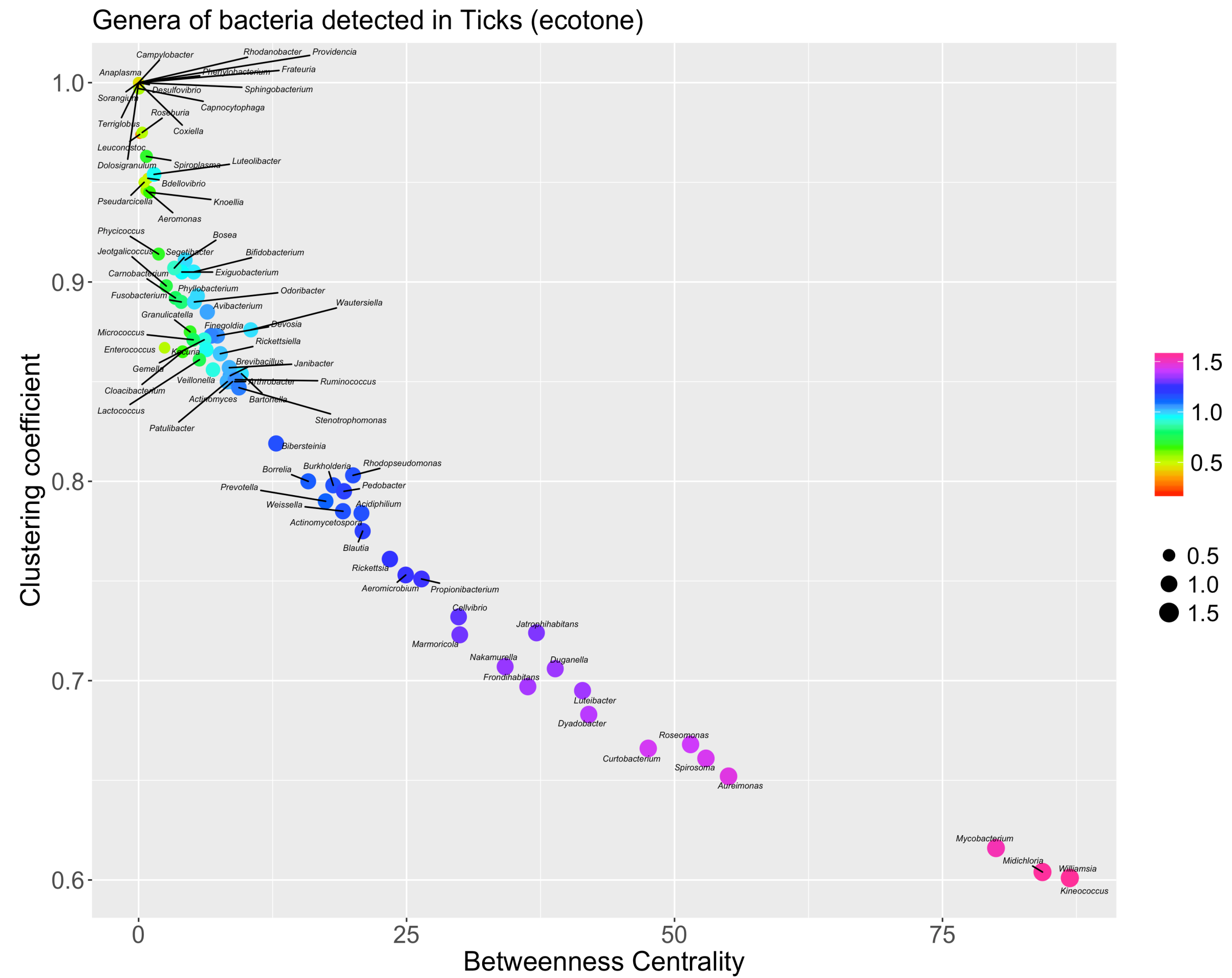

C

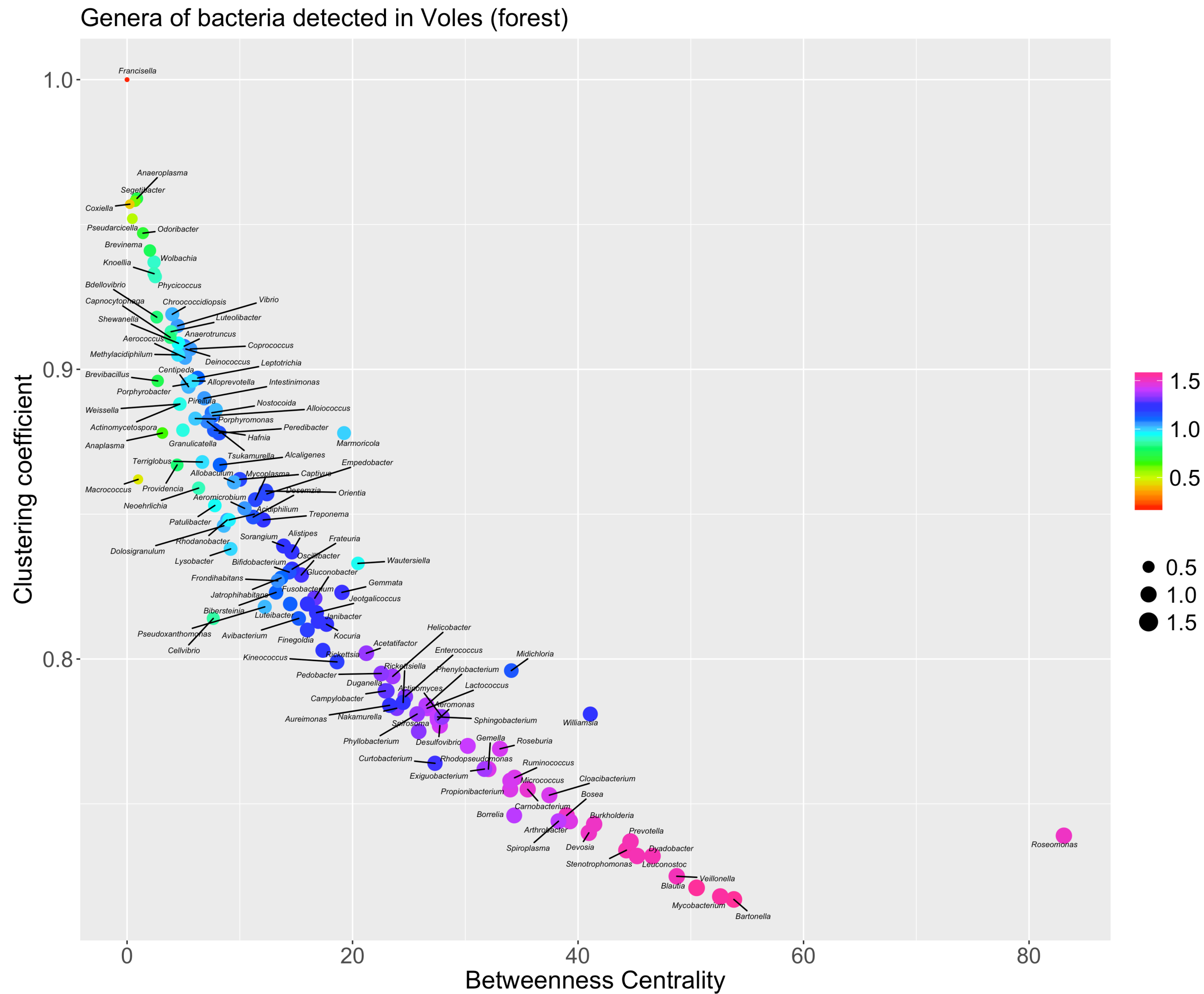

D

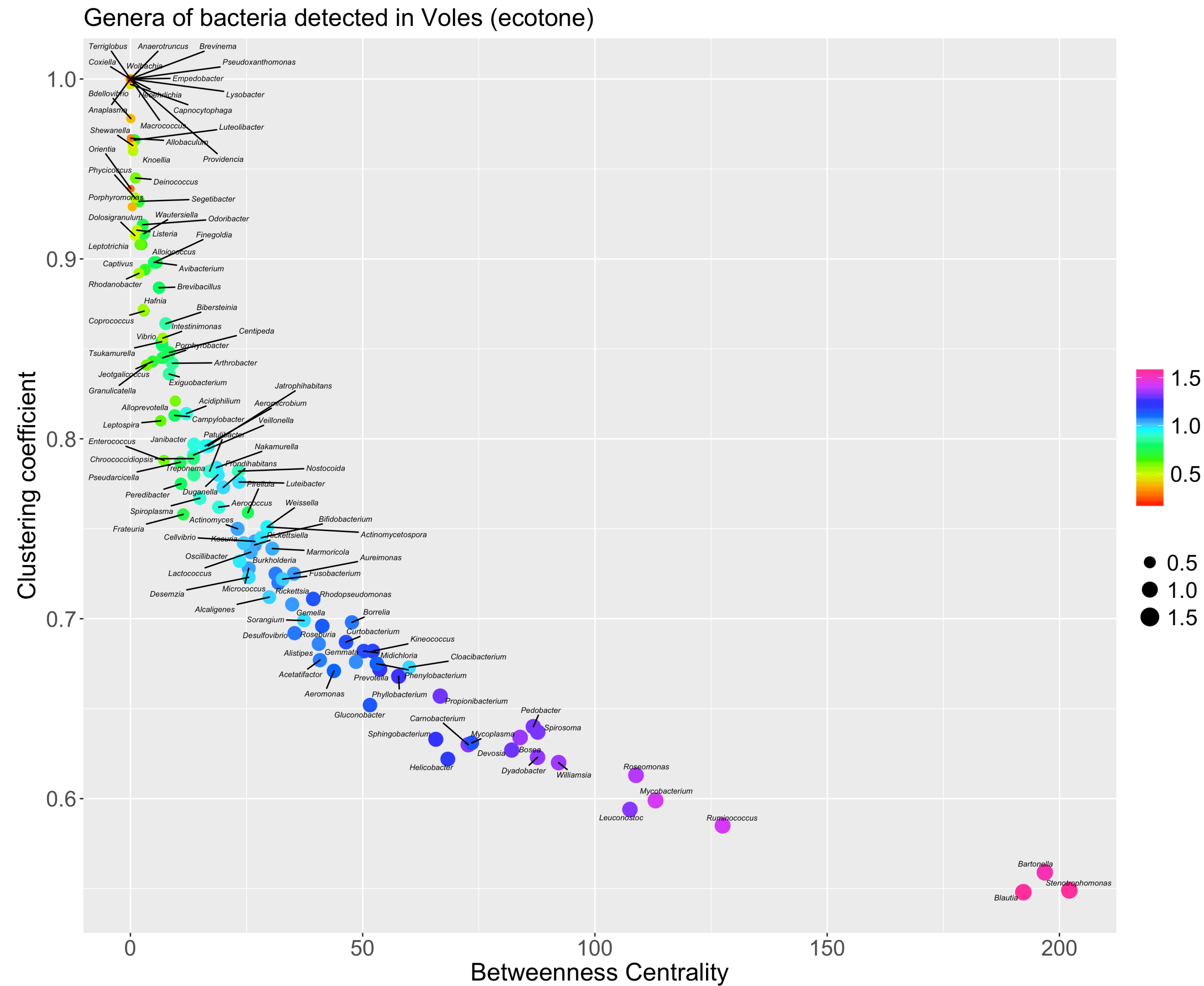

Supplement: Figure S7 — The relationships between Betweenness Centrality (BNC), Clustering coefficient (CC) and PageRank in the networks of co-occurring bacteria in ticks-forest (A), ticks-ecotone (B), voles-forest (C), and voles-ecotone (D). Each plot is colored and sized according to the values of PageRank and placed in the intersection of values of BNC and CC. The information is the same as in Figure 2 but including labels for all the bacteria. [file Image_7.PDF]
