# Supplementary material for: High Throughput Sequencing and Network Analysis Disentangle the Microbial Communities of Ticks and Hosts Within and Between Ecosystems
Source: Front Cell Infect Microbiol. 2018 Jul 9;8:236. doi: 10.3389/fcimb.2018.00236 (PMC6046413; doi:10.3389/fcimb.2018.00236)
Supplement: Figure S2 — The figure displays a guide to the application of network analysis to microbiome studies. Firstly, co-occurrence (i.e., the number of times a genus co-occurs with other genera of bacteria) of microbial taxa among the samples is calculated. The co-occurrence table is used as input for network analysis software (A). The co-occurrence table is used to calculate important indexes including the Degree, Centrality and Modularity of the network. The software provides a plain and basic visual depiction of the network (B) that can be enhanced by coloring the communities of nodes and their links, and resizing each circle representing the microorganism by a value, in this case the Centrality (C). Phylogenetic tree of bacterial genera is built using evolutionary distances calculated from pairwise comparisons of full length 16S DNA sequences available for bacteria species within each genus (D). This tree can be used to calculate the phylogenetic distance among the taxonomic ranks of bacteria, track the phylogenetic signal (using Pagel's λ) of the indexes of the networks and evaluate the phylogenetic composition of the communities of co-occurring bacteria (D). [file Image_2.PDF]

Data on co-occurrence of bacteria

A

	**genus 1**	**genus 2**	**genus 3**	**genus 4**	**genus n**
**genus 1**					
**genus 2**	18				
**genus 3**	22	1			
**genus 4**	1	22	0		
**genus n**	32	5	4	67	

- Degree
- Indexes of Centrality
- Modularity

Species degree: the number of different species a certain species interacts with.
Degree distribution: the frequency distribution of the number of interactions per species.
Centrality: the relative importance of each microorganism in the context of the network.
Modularity: the group of microorganisms that co-occur most frequently among them than with other microorganisms (clusters)

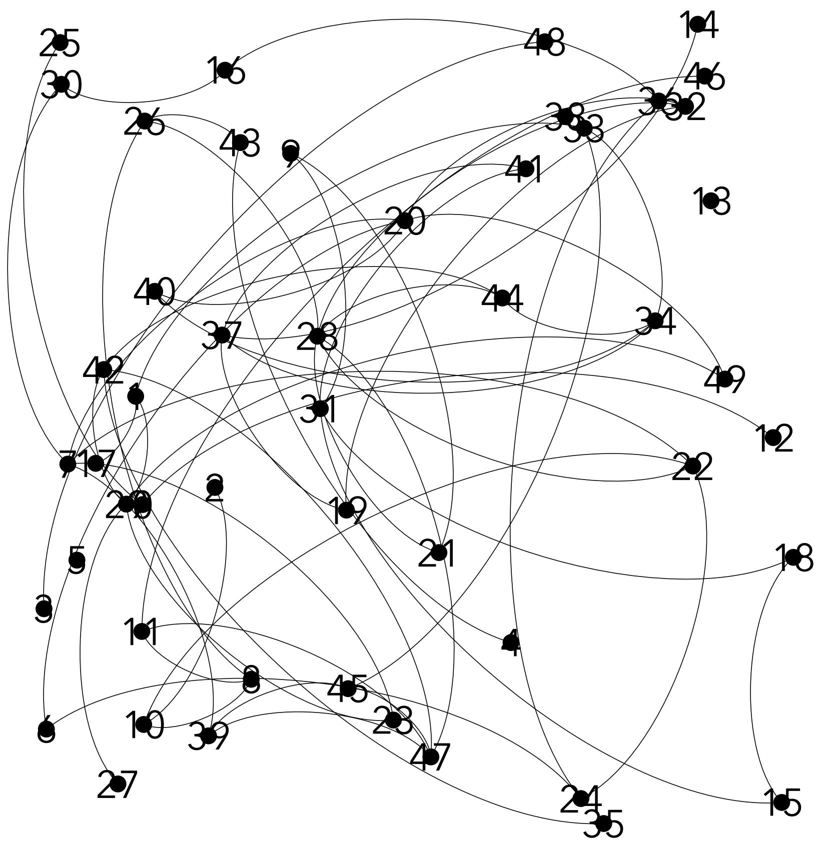

B

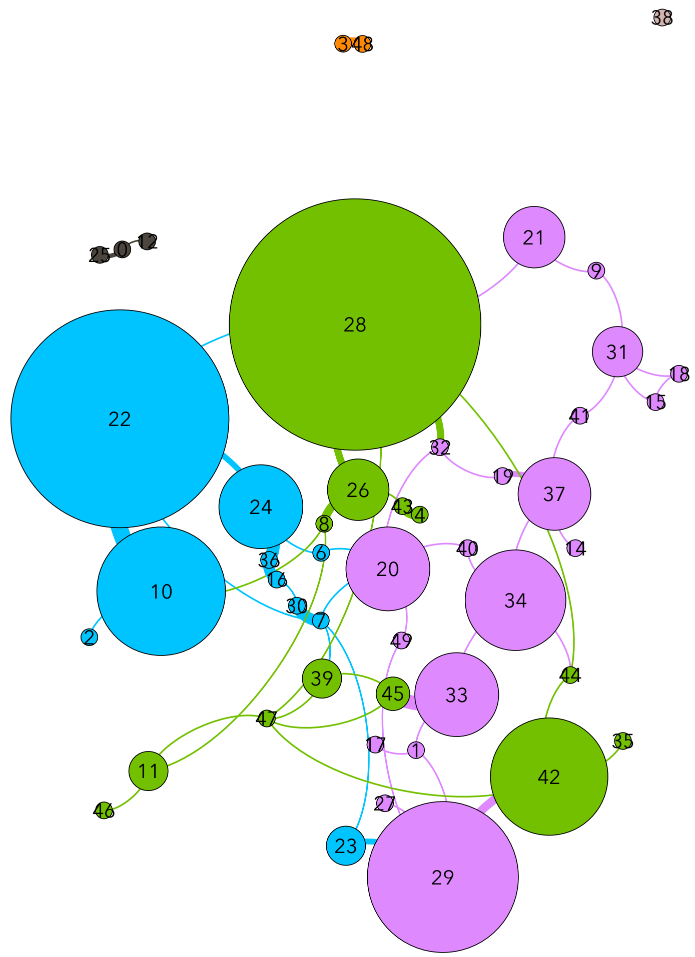

C

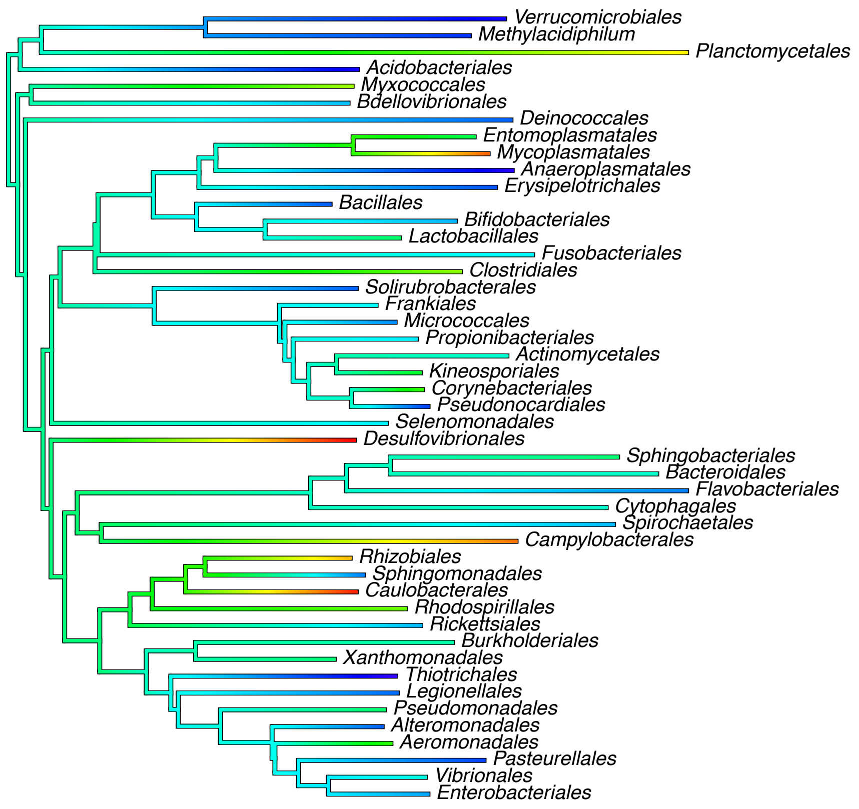

D

Phylogenetic distance
Pagel's λ
