# Supplementary material for: High Throughput Sequencing and Network Analysis Disentangle the Microbial Communities of Ticks and Hosts Within and Between Ecosystems
Source: Front Cell Infect Microbiol. 2018 Jul 9;8:236. doi: 10.3389/fcimb.2018.00236 (PMC6046413; doi:10.3389/fcimb.2018.00236)
Supplement: Figure S8 — The log-ratio of the number of reads of each genus of bacteria (voles/ticks or ticks/voles) plotted along the range of weights of each genus in the four networks of co-occurring bacteria. The color and the size of each dot and the size of the label of the genus show the log-ratio between the number of reads. The information is the same as in Figure 3 but including labels for all the bacteria. (A) voles/ticks, forest; (B) voles/ticks, ecotone; (C) ticks/voles, forest; (D) ticks/voles, ecotone. [file Image_8.PDF]

Ratio voles/ticks of co−occurrence of genera of bacteria (forest)

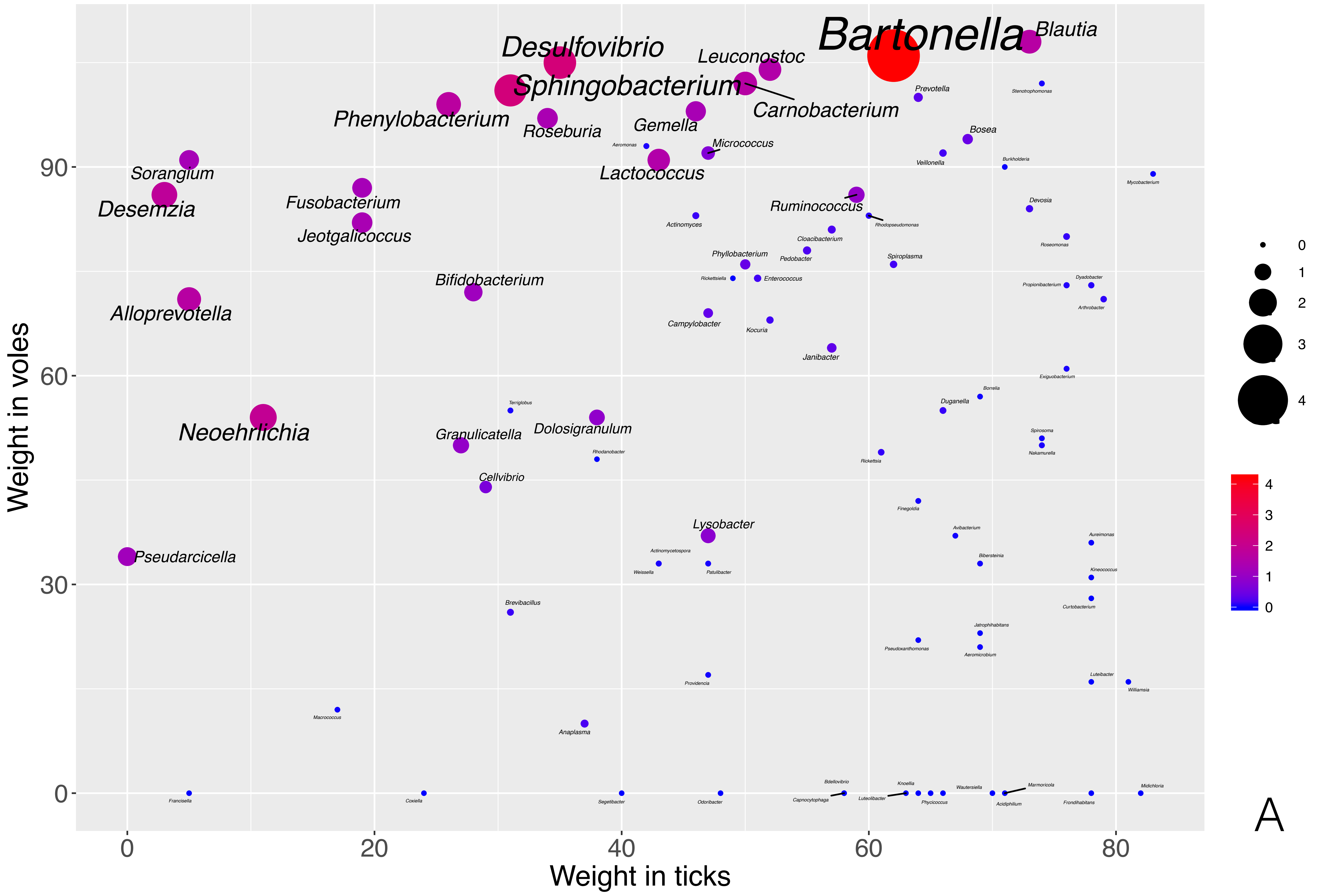

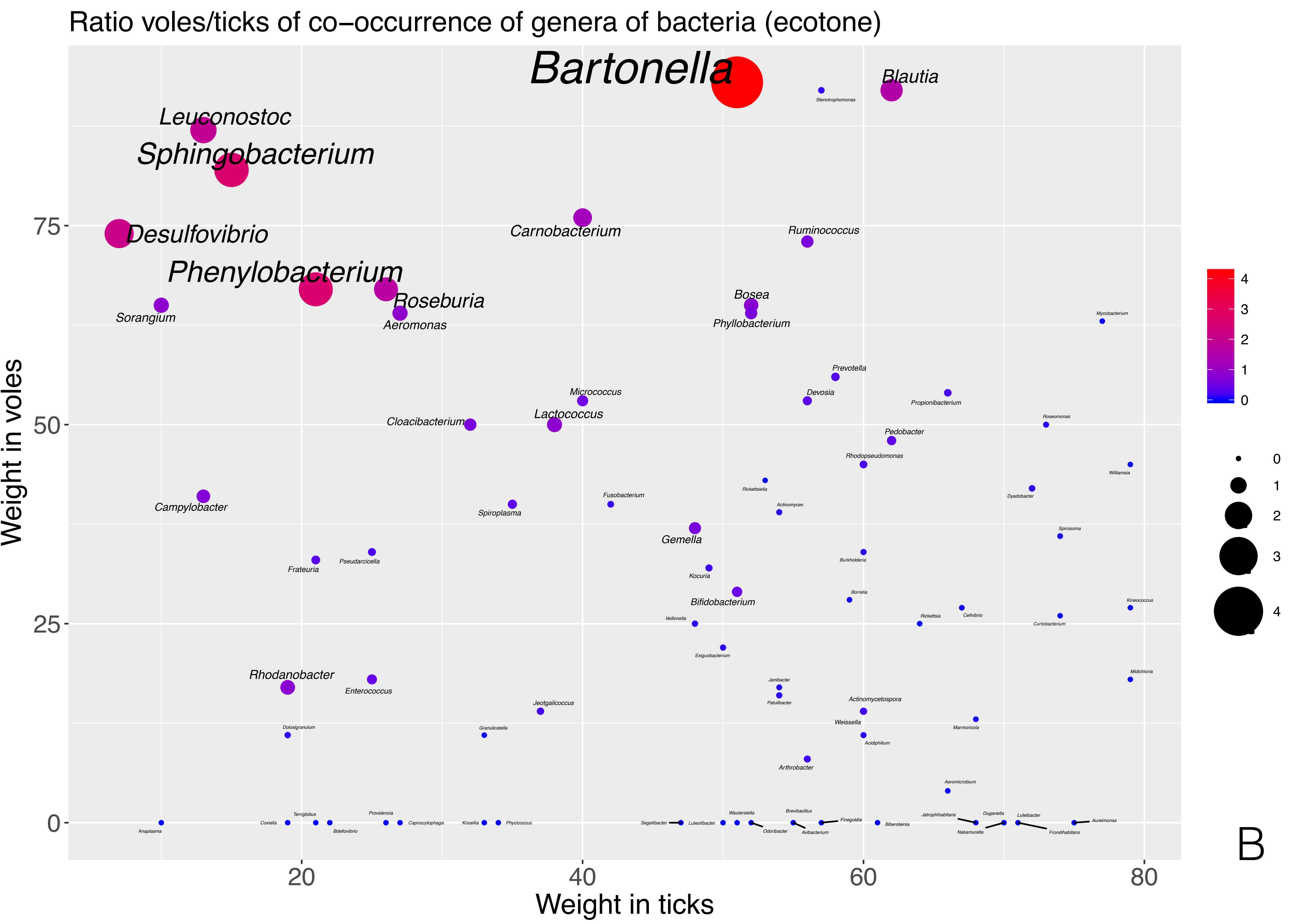

Ratio voles/ticks of co−occurrence of genera of bacteria (ecotone)
Weight in voles
Weight in ticks
0
25
50
75
20
40
60
80
Bartonella
Blautia
Stenotrophomonas
Leuconostoc
Sphingobacterium
Desulfovibrio
Carnobacterium
Ruminococcus
Phenylobacterium
Roseburia
Sorangium
Aeromonas
Bosea
Phyllobacterium
Mycobacterium
Prevotella
Devosia
Propionibacterium
Micrococcus
Lactococcus
Cloacibacterium
Roseomonas
Pedobacter
Rhodopseudomonas
Williamsia
Rickettsiella
Dyadobacter
Campylobacter
Spiroplasma
Fusobacterium
Actinomyces
Spirosoma
Gemella
Frateuria
Pseudarcicella
Burkholderia
Kocuria
Bifidobacterium
Borrelia
Kineococcus
Veillonella
Rickettsia
Cellvibrio
Curtobacterium
Exiguobacterium
Midichloria
Rhodanobacter
Enterococcus
Janibacter
Patulibacter
Jeotgalicoccus
Actinomycetospora
Weissella
Marmoricola
Dolosigranulum
Granulicatella
Acidiphilium
Arthrobacter
Aeromicrobium
Anaplasma
Coxiella
Terriglobus
Bdellovibrio
Providencia
Capnocytophaga
Knoellia
Phycicoccus
Segetibacter
Luteolibacter
Wautersiella
Odoribacter
Brevibacillus
Avibacterium
Finegoldia
Bibersteinia
Jatrophihabitans
Nakamurella
Duganella
Luteibacter
Frondihabitans
Aureimonas
4
3
2
1
0
0
1
2
3
4
B

Ratio ticks/voles of co−occurrence of genera of bacteria (forest)

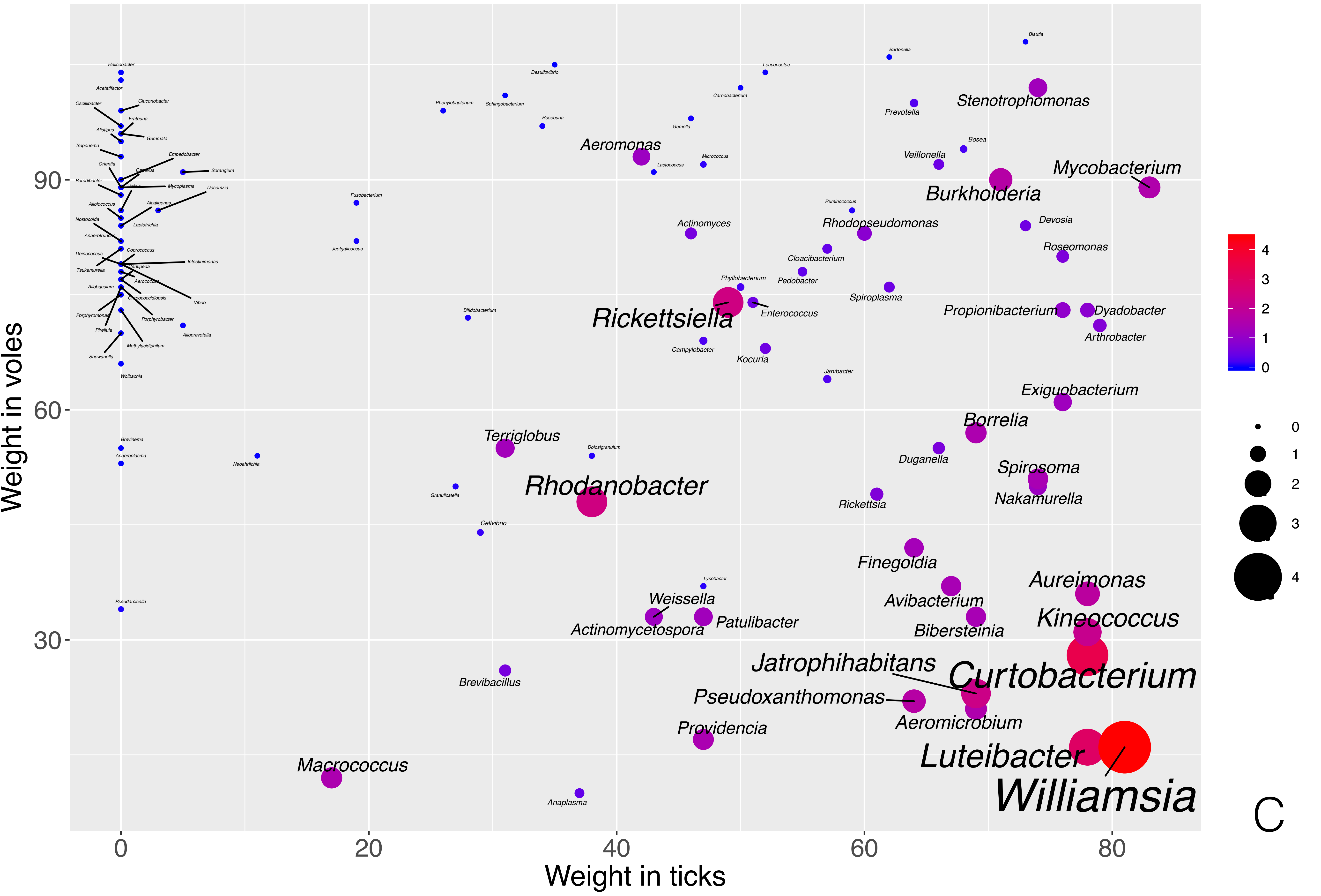

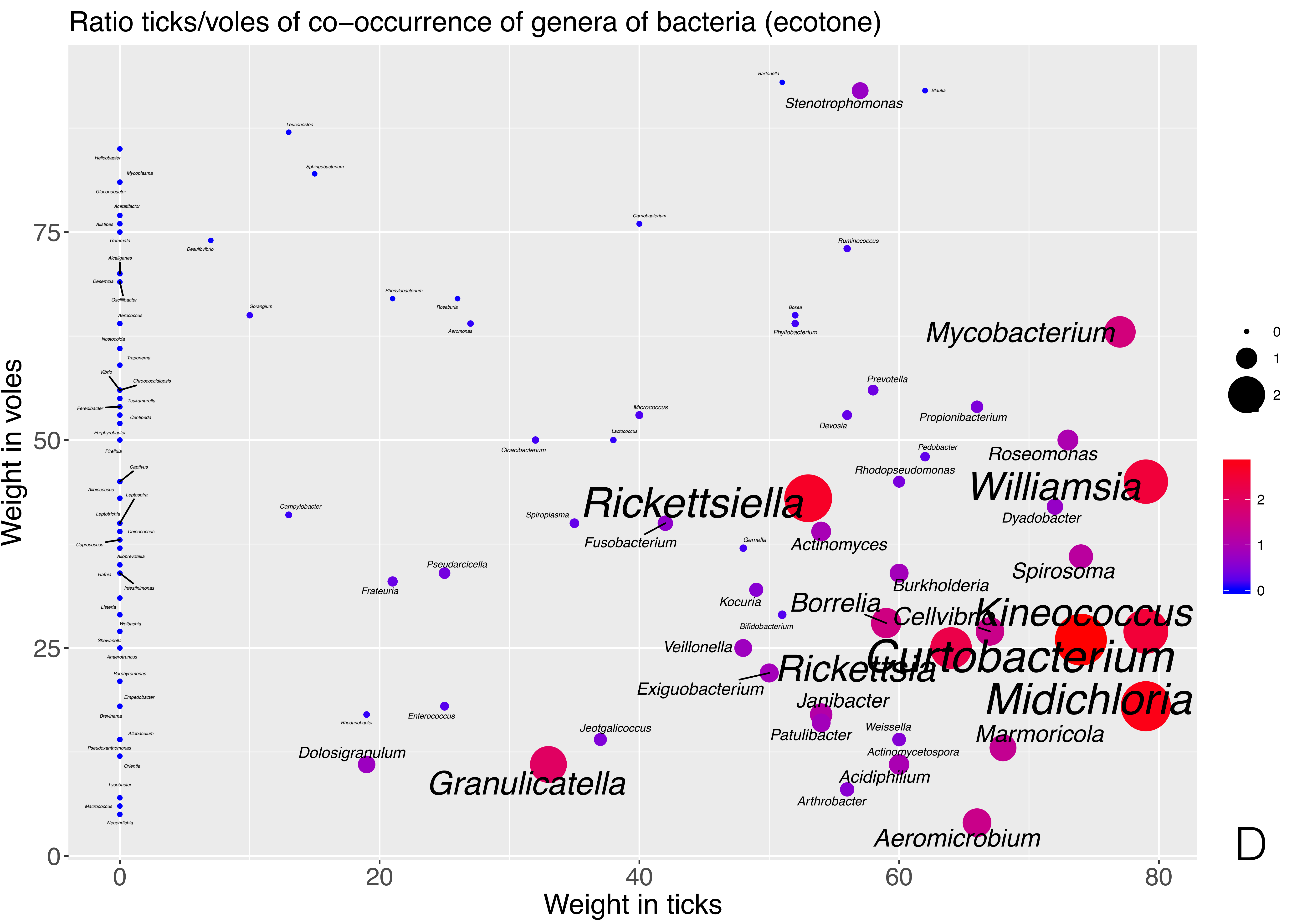
Ratio ticks/voles of co−occurrence of genera of bacteria (ecotone)
Weight in voles
Weight in ticks
0
25
50
75
0
20
40
60
80
0
1
2
0
1
2
D
Bartonella
Blautia
Stenotrophomonas
Leuconostoc
Helicobacter
Mycoplasma
Sphingobacterium
Gluconobacter
Acetatifactor
Alistipes
Gemmata
Desulfovibrio
Carnobacterium
Ruminococcus
Alcaligenes
Desemzia
Oscillibacter
Phenylobacterium
Roseburia
Sorangium
Aerococcus
Aeromonas
Bosea
Phyllobacterium
Mycobacterium
Nostocoida
Treponema
Vibrio
Chroococcidiopsis
Tsukamurella
Peredibacter
Centipeda
Prevotella
Microcoecus
Propionibacterium
Devosia
Porphyrobacter
Lactococcus
Cloacibacterium
Pirellula
Pedobacter
Roseomonas
Captivus
Rhodopseudomonas
Alloiococcus
Leptospira
Williamsia
Leptotrichia
Campylobacter
Rickettsiella
Spiroplasma
Dyadobacter
Deinococcus
Coprococcus
Fusobacterium
Gemella
Actinomyces
Alloprevotella
Hafnia
Intestinimonas
Pseudarcicella
Spirosoma
Frateuria
Burkholderia
Listeria
Kocuria
Borrelia
Kineococcus
Wolbachia
Bifidobacterium
Cellvibrio
Shewanella
Veillonella
Curtobacterium
Anaerotruncus
Porphyromonas
Rickettsia
Exiguobacterium
Empedobacter
Janibacter
Midichloria
Brevinema
Rhodanobacter
Enterococcus
Jeotgalicoccus
Weissella
Allobaculum
Patulibacter
Marmoricola
Pseudoxanthomonas
Dolosigranulum
Actinomycetospora
Orientia
Granulicatella
Acidiphilium
Lysobacter
Macrococcus
Arthrobacter
Neoehrlichia
Aeromicrobium
